# Multi‐omic integration of DNA methylation and gene expression data reveals molecular vulnerabilities in glioblastoma

**DOI:** 10.1002/1878-0261.13479

**Published:** 2023-07-20

**Authors:** Pablo Santamarina‐Ojeda, Juan Ramón Tejedor, Raúl F. Pérez, Virginia López, Annalisa Roberti, Cristina Mangas, Agustín F. Fernández, Mario F. Fraga

**Affiliations:** ^1^ Health Research Institute of Asturias (ISPA) Spain; ^2^ Foundation for Biomedical Research and Innovation in Asturias (FINBA) Spain; ^3^ University Institute of Oncology of Asturias (IUOPA) Spain; ^4^ Centre for Biomedical Network Research on Rare Diseases (CIBERER) Madrid Spain; ^5^ Nanomaterials and Nanotechnology Research Centre (CINN‐CSIC) Principality of Asturias Spain

**Keywords:** DNA methylation, gene expression, glioblastoma, mesenchymal, multi‐omics, patient‐derived GBM stem cells

## Abstract

Glioblastoma (GBM) is one of the most aggressive types of cancer and exhibits profound genetic and epigenetic heterogeneity, making the development of an effective treatment a major challenge. The recent incorporation of molecular features into the diagnosis of patients with GBM has led to an improved categorization into various tumour subtypes with different prognoses and disease management. In this work, we have exploited the benefits of genome‐wide multi‐omic approaches to identify potential molecular vulnerabilities existing in patients with GBM. Integration of gene expression and DNA methylation data from both bulk GBM and patient‐derived GBM stem cell lines has revealed the presence of major sources of GBM variability, pinpointing subtype‐specific tumour vulnerabilities amenable to pharmacological interventions. In this sense, inhibition of the AP‐1, SMAD3 and RUNX1/RUNX2 pathways, in combination or not with the chemotherapeutic agent temozolomide, led to the subtype‐specific impairment of tumour growth, particularly in the context of the aggressive, mesenchymal‐like subtype. These results emphasize the involvement of these molecular pathways in the development of GBM and have potential implications for the development of personalized therapeutic approaches.

## Graphical abstract

The integration of epigenomic and transcriptional data in the context of multi‐omics factor analysis has revealed potential molecular vulnerabilities in glioblastoma (GBM). A set of functional experiments and pharmacological interventions on the most prominently altered pathways in a patient‐derived GBM stem cell model illuminates the role of these factors as potential therapeutic targets for future interventions.

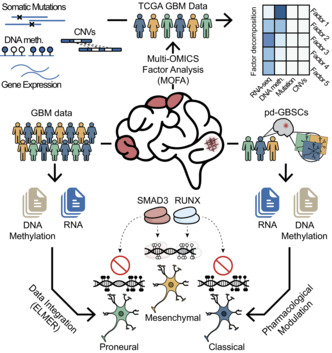

**Abbreviations**
CL: classical
CNS: central nervous system
CNV: copy number variation
DEG: differentially expressed gene
DMP: differentially methylated position
ELMER: enhancer linking by methylation/expression relationships
FDR: false discovery rate
GBM: glioblastoma
G‐CIMP: glioma‐CpG island methylator phenotype
GSEA: gene set enrichment analysis
GTRD: gene transcription regulation database
HGCC: human glioblastoma cell culture
IDH: isocitrate dehydrogenase
MGMT: methylguanine methyltransferase
MOFA: multi‐omics factor analysis
MS: mesenchymal
OR: odds ratio
Pd‐GBSCs: patient‐derived GBM stem cells
PN: proneural
RRBS: reduced‐representation bisulfite sequencing
TCGA: The Cancer Genome Atlas
TF: transcription factor
TFBS: transcription factor binding sites
TGF‐β1: transforming growth factor beta 1
UMAP: uniform manifold approximation and projection
VST: variance stabilizing transformation
WES: whole exome sequencing
WT: wild type

## 1 Introduction

Central nervous system (CNS) tumours encompass all the malignancies derived from the brain and spinal cord, and are currently being diagnosed according to both histological and molecular parameters [1]. Within the diffuse gliomas group, glioblastoma (GBM) is considered the most common and lethal tumour in adults and is characterized by a poor prognosis and an average survival of 8 months, regardless of treatment, although medical interventions including surgical resection/radio‐ and chemotherapy may slightly increase survival time [2]. Despite recent advances in knowledge of the molecular mechanisms underlying GBM tumourigenesis and progression, efforts to extend patient survival remain a challenge, with deep tumoural heterogeneity, elevated tumoural infiltration and the presence of stem cell‐like tumoural cells being the main sources of the aggressive behaviour of GBM and relapse after first‐line treatment [3, 4].

The development of novel multi‐omic technologies is undergoing continuous sophistication, leading to ever‐more accurate methods for identifying and stratifying brain tumours, thus improving disease management and advancing antitumoural therapy. Molecular approaches have been demonstrated to be valuable tools for classifying these patients in terms of prognosis and tumoural progress: e.g., profiling the mutational status of *IDH1* and *IDH2* genes [5], since patients bearing mutations in these genes have a better prognosis than those with the wild‐type phenotype [1, 6, 7]. Taking a step forward, transcriptional approaches with bulk tumours have led to the stratification of *IDH* ^WT^ GBMs into three transcriptional subtypes: proneural (PN), mesenchymal (MS) and classical (CL), revealing differences in their average survival, with the MS subtype having the worst prognosis [8, 9, 10]. Furthermore, the results of recent single‐cell RNA‐seq experiments on *IDH* ^WT^ patients with GBM show strong similarities with previous classifications based on bulk tumours [10, 11, 12]. In addition, information circumscribed in the epigenetic layer, such as DNA methylation, has emerged as an effective molecular tool to discriminate between patients with GBM. Global methylation studies based on microarrays and reduced representation bisulfite sequencing (RRBS) [13, 14] have also provided equivalent as well as innovative insights into defining GBM subtypes, since methylation‐based classifications faithfully resemble the previous transcriptomic‐based subtypes and have contributed to defining and separating patient groups that have the hypermethylation phenotype (G‐CIMP) as the main driver, which is in line with patient categorization according to *IDH* status [6, 15]. Efforts to investigate the interplay between gene expression and epigenetic profiles have provided a window of opportunity both for refining the methods used to stratify patients with GBM and for spotting non‐described subtype‐associated cancer vulnerabilities in a more precise way.

In recent years, refinements of cancer research models have increased the accuracy of new experimental approaches, leading to a more straightforward understanding of tumour biology. In this regard, patient‐derived cell culture models, grown under neural stem conditions, are widely considered to be faithful models that mimic specific cellular programmes of the original tumour and, eventually, their clinical responses [16, 17, 18, 19, 20]. In this study, we have performed genome‐wide transcriptomic and DNA methylation analyses in both bulk GBM and patient‐derived GBM stem cell (pd‐GBSC) cultures from different GBM subtypes with the aim of uncovering subtype‐specific vulnerabilities within GBM *IDH* ^WT^ tumours and identifying potential molecular pathways amenable to therapeutic interventions. We have performed a comprehensive, subtype‐specific analysis of these multi‐omic layers with the aim of interpreting the tumoural landscape of GBM from a broader perspective. Subsequent transcriptomic and epigenomic data integration has allowed us to reveal subtype‐specific molecular pathways governed by AP‐1, SMAD3 and RUNX1/RUNX2 transcription factors (TF) in both pd‐GBSCs and bulk tumours. The biological effects of the identified subtype‐associated TFs were assessed by means of pharmacological interventions, verifying their implications for tumour sustainability in the most aggressive MS‐like GBM subtype. These results highlight the potential of multi‐omic approaches for the discovery of cancer cell vulnerabilities and provide a set of therapeutic targets that could be employed in the development of novel, customized therapies in the context of GBM.

## 2 Materials and methods

### 2.1 Multi‐omics factor analysis of GBM data

Multi‐omics factor analysis models were performed using the r/bioconductor package mofa2 (v1.0.1) [21]. Batch corrected, paired DNA methylation microarrays (Illumina 27 K/450 K, San Diego, CA, USA), RNA‐seq (Illumina HiSeq), mutation (exome‐seq) and copy number variation (CNV, Affymetrix Human SNP Array 6.0) data from GBM samples generated by the TCGA (The Cancer Genome Atlas) consortium [8] were obtained from the ucsc XenaBrowser TCGA‐GBM Data Hub (https://xenabrowser.net/). Sample purity was assessed using the estimate tool [22] and those samples with a purity score below 0.70, as well as samples including *IDH* mutations and those corresponding to the G‐CIMP cluster were discarded for downstream analyses. RNA‐seq data counts were scaled using the variance stabilizing transformation (VST) approach (fitType = local) from the r/bioconductor package deseq2 (v.1.30.1) [23]. The DNA methylation matrix was scaled up to a normal distribution using a logarithmic approach. To improve model accuracy, an additional feature selection approach was performed to select the most informative features in the datasets of DNA methylation, gene expression and mutations. We selected the top 2% of the most variable CpG sites from the DNA methylation dataset (4074 features), the top 50% of the most variable genes in terms of their Wang GBM signature [10] from the RNA‐seq data (3153 features), the 45 most informative mutations and the 75 most informative CNVs identified in patients with TCGA GBM. CNV data were scaled to −1 or +1 values to represent copy number losses or gains, respectively. Different MOFA models, from 1 to 25 factors, were initialized to find the optimal number of factors, and a final MOFA model with 5 factors was selected based on the ‘elbow method’ applied to the evidence lower bound and the total variance explained of TCGA data (Fig. S1). Additional MOFA parameters were set to: convergence mode (slow), seed (1) and likelihoods (RNA‐seq: gaussian; DNA methylation: gaussian; mutation: Bernoulli; CNV: gaussian). MOFA clusters were obtained using spectral clustering on the MOFA UMAP representation using the top three most informative factors (each of which had an explained variance greater than 10%), and the r/bioconductor package kernlab (v.0.9.29) with the following parameters: centres = 3, kerner = rbfdot, iterations = 10 000. Survival analyses were performed with the Kaplan–Meier plots using the resulting sample stratification of the different MOFA clusters and were generated with the r/cran packages survival (v.3.2.13) and survminer (v.0.4.9). *P*‐values indicated significant differences (*P*‐value < 0.05) in event rates between the different cluster groups or significant associations between MOFA factors with survival time and were calculated using the log‐rank test approach or Cox proportional‐hazard models, respectively. The methylation status of the *MGMT* promoter was estimated using the r package mgmtstp27 (v.0.6‐4) [24].

### 2.2 Human brain samples and patient‐derived GBM stem cell lines

Non‐tumoural human brain cortex (*n* = 4) was obtained from the biobank of the Principality of Asturias (Hospital Universitario Central de Asturias, Oviedo, Spain). These brains showed no signs of either neurodegeneration or histological alterations. All the patients gave written informed consent, and all experimental and ‐omics procedures were performed in accordance with the standards established by the Declaration of Helsinki and were approved by the Clinical Research Ethics Committee of the Principality of Asturias (Project ID: 102/19). Patient‐derived glioblastoma stem cells from distinct GBM subtypes (*n* = 9, RRIDs: CVCL_IR79 (U3047), CVCL_IR90 (U3071), CVCL_IS00 (U3117), CVCL_IR61 (U3013), CVCL_IR91 (U3073), CVCL_IR65 (U3020), CVCL_IR84 (U3056), CVCL_IR63 (U3017), CVCL_IR76 (U3039)) were acquired from primary GBM samples obtained from the Human Glioblastoma Cell Culture (HGCC, https://www.hgcc.se) resource, which have been previously described and validated [20, 25]. These pd‐GBSCs were purchased in 2020 from the Department of Immunology, Genetics and Pathology of the Uppsala University (Uppsala, Sweden, HGCC resource) and were authenticated using multiplex short tandem repeat (STR) profiling as described by Xie et al. [20]. All pd‐GBSC samples were obtained in accordance with protocols approved by a regional ethics committee in Sweden. Categorization into different GBM subtypes – PN, MS and CL – according to the classification proposed by Verhaak et al. [9] was carried out by the HGCC resource using Affymetrix GeneChip Human Exon 1.0 ST arrays [20].

### 2.3 Cell culture procedures

pd‐GBSCs were cultivated under stem cell conditions following the procedure described in [18] in order to retain as many of the original tumour features as possible. Cell expansion and subsequent experiments were conducted in Dulbecco's Modified Eagle Medium (DMEM/F12, Cat. No: 1331020, Gibco, Waltham, MA, USA) supplemented with N2 (100×: 25 μg·mL^−1^ insulin (Cat. No: I6634, Sigma, Darmstadt, Germany)), 100 μg·mL^−1^ apo‐transferrin (Cat. No: 11108016, Gibco), 50 μg·mL^−1^ bovine serum albumin fraction V 7.5% (Cat. No: 15260037, Gibco), 0.02 μg·mL^−1^ progesterone (Cat. No: P8783, Sigma), 16 μg·mL^−1^ putrescine (Cat. No: P5780, Sigma) and 5.18 ng·mL^−1^ sodium selenite (Cat. No: S5261, Sigma), 1× B‐27 (Cat. No: 17504044, Gibco), 1 mm sodium pyruvate (Cat. No: S8636, Sigma), 500 units·mL^−1^ penicillin/streptomycin (Cat. No: P4333, Sigma) and supplemented daily with 10 ng·mL^−1^ EGF (Cat. No: 78006.1, Stem Cell Tech., Vancouver, BC, Canada), and 10 ng·mL^−1^ FGF‐2 (Cat. No: 78003, Stem Cell Tech). Cells were cultivated in flasks and plates previously coated with 10 μg·mL^−1^ laminin (Cat No: L2020, Sigma) for 4 h at 37 °C. Cell cultures were incubated at 37 °C in a humidified atmosphere with 5% CO_2_ and were routinely checked to ensure they were mycoplasma‐free (Mycoplasma Gel Detection Kit, Cat. No: 90.022‐4544, Biotools, Madrid, Spain).

### 2.4 Nucleic acid purification for high‐throughput assays

Genomic DNA was purified from non‐tumoural brains and pd‐GBSCs using 0.5% SDS Lysis buffer (100 mm NaCl, 20 mm Tris pH: 8.0, 25 mm EDTA pH: 8.0, 0.5% SDS) and 1 mg·mL^−1^ Proteinase K (Cat. No: 03115801001, Roche, Basel, Switzerland) for 4 h to lysate the samples. This was followed by a standard phenol‐chloroform protocol for phase separation and ethanol extraction as the precipitation step. Total RNA from the same set of samples was extracted using the RNeasy Mini Kit (Cat. No: 74104, QIAGEN, Hilden, Germany) following the manufacturer's instructions. DNA and RNA integrity and quantity were determined by spectrophotometry using the Nanodrop system (Nanodrop1000, ThermoFisher, Waltham, MA, USA) and verified with the Qubit™ fluorometer system (Qubit™ 4 fluorometer, ThermoFisher).

### 2.5 Gene expression analyses

Next‐generation RNA sequencing of non‐tumoural brains and pd‐GBSC lines was performed by Azenta Life Sciences. mRNA libraries were prepared using the Illumina Poly(A) selection method and the NEBNext ultra‐RNA sequencing Library prep kit. Sequencing libraries were clustered in an Illumina Novaseq Instrument and sequenced using a 2 × 150 bp paired‐end (PE) configuration. In addition, we obtained raw RNA sequencing FASTQ files corresponding to GBM samples from a recent glioblastoma study [17] and we imputed the GBM subtype using the gene expression‐based classification of Wang et al. [10] via the GlioVis portal [26], obtaining an average of 34.4 m (16.9–62.5 m) PE reads per sample across all datasets. We used fastp (v.20.1) [27] for adapter removal, and the subsequent filtered reads were aligned to the human GRCh38 genome using rsem (v.1.3.1) [28] including the hisat2‐hca PE mode. Overall mapping efficiency was 69.2% (48.1–86.34) for all the samples analysed. We estimated the counts per gene using the RSEM output and the tximport function of the r/bioconductor package tximport (v.1.18.0) [29]. Low‐abundance genes with less than 10 counts (per sample) or less than 200 counts (all samples) were discarded for downstream analyses. Differential gene expression analyses between control samples and the different GBM subtypes were performed using the r/bioconductor package deseq2 (v.1.30.1) [23]. The resulting *P*‐values were corrected using the Benjamini and Hochberg method, and we considered a threshold of adj. *P*‐value < 10^−6^ to indicate statistical significance in regard to differentially expressed genes (DEGs). Subsequent downstream analyses were performed using the normalized gene expression matrix (VST approach). The coefficient of variation for GBM and pd‐GBSCs lines was performed following Tejedor et al. [30].

### 2.6 Gene set enrichment and modular gene co‐expression analyses

Gene set enrichment analyses were performed as described elsewhere [30] with gsea (v.4.0.3) [31], using VST normalized gene expression matrices and the C2 (v.5.2), C5 (v.5.2) and hallmark (v.5.2) MSigDB collections [32]. Modular co‐expression analyses were performed with the cemitool package (v.1.14.0) [33]. A prior gene variance filtering step was introduced (*P*‐value < 0.05), and additional parameters were set up for proper clustering purposes (correlation type = Spearman, diss_threshold = 0.7). Over‐representation analyses (ORAs) of the biological functions associated with each of the modules were performed with the mod_ora function (*P*‐value < 0.001) using the C2, the C5 and the hallmark gene sets from the MSigDb database.

### 2.7 Microarray‐based DNA methylation analyses

Genomic DNA samples were submitted to Cambridge Genomic Services for microarray‐based DNA methylation profiling to be performed using the Infinium HumanMethylationEPIC 850 K Beadchip platform (Illumina). Raw IDAT files were processed using the r/bioconductor package minfi (v.1.22.1) [34]. Data were normalized using the ssNOOB algorithm with the default parameters (offset = 15, dyeCorr = TRUE and dyeMethod = “single”) and probe type distribution was corrected using the BMIQ approach [35]. Probes overlapping genetic variants, probes located in sex chromosomes, cross‐reactive and multimapping probes, and probes with at least one sample with a detection *P*‐value > 0.01 were discarded for downstream purposes. Resulting M‐values were used for differential methylation analyses, assuming homoscedasticity, while B‐values were used for graphical visualization and correlation analyses between DNA methylation and gene expression subtypes (see below). In order to perform accurate differential methylation analyses, a surrogate variable analysis (SVA) [36] was employed to account for possible batch effects or confounding variables. Detected surrogate variables (SVs) were added to the phenotypical data and included in the definition of the model in order to detect differentially methylated probes (DMPs). DMP identification was determined by the moderated *t*‐test implemented in the r/bioconductor package limma (v.3.38.3) [37]. The resulting *P*‐values were corrected for multiple testing using the Benjamini–Hochberg method (FDR). An FDR threshold of 0.05 and a minimum absolute difference of 0.3 between mean DNA methylation B‐values of different GBM subtypes and controls were required to determine significant DMPs.

### 2.8 CpG status and region set enrichment analyses

DMPs were assigned to their corresponding genomic context or genomic location using, respectively, the r/bioconductor packages illuminahumanmethylationepic.anno.ilm10b4.hg19 (v.0.6.0) and chipseeker (v.1.18.0) [38]. Chromatin enrichment analyses were performed with the r/bioconductor package lola (v.1.4.0) [39]. DMP enrichments in chromatin state data from 10 neural‐related epigenomes were obtained from NIH Roadmap's ChromHMM expanded 18‐state model [40]. Additional DMP enrichments in transcription factor binding sites (TFBSs) were performed using data from human meta‐clusters obtained from the GTRD database [41] as described in [30]. The enrichment significance of the abovementioned analyses was calculated using one‐sided Fisher's tests (adj. *P*‐value < 0.05), comparing the overlap of DMPs with the dataset of interest and using the set of filtered probes from the HumanMethylationEPIC as background.

### 2.9 Integration of DNA methylation and gene expression data

Integration of DNA methylation (B‐values) and gene expression (Log_2_ + 1 TPM) data were performed using the r/bioconductor package elmer (v.2.6.3) [42]. DMPs identified in the different GBM subtypes were paired with expression data of their 10 most proximal genes in order to identify functional connections between the methylation status of a given region and their potential transcriptional targets. Paired DNA methylation loci‐gene expression targets were identified using the supervised mode of the get.pair function (permutation size = 10 000, Pe = 0.05) for either hyper‐ or hypomethylated probes. Identification of significantly enriched TF motifs in the correlated gene‐DMP pairs was obtained using the get.enriched.motif function from the elmer package with the following parameters: minimum incidence = 10, lower odds ratio (OR) = 1.1 and minimum motif quality = B. In order to focus on the most significant gene‐DMP pairs, a subsequent stringent filtering approach was applied to absolute values so as to retain only those gene‐DMP Spearman correlations greater than 0.5 and with greater than 2‐fold gene expression changes (Log_2_FC).

### 2.10 Subcellular fractionation and immunoblotting

Subcellular fractionation of U3117‐PN, U3073‐MS and U3056‐CL cells was performed according to the protocol described by Dimauro et al. [43]. Protein extracts from purified nuclear and cytosolic fractions were quantified using the Pierce™ BCA Protein Assay kit (Cat. No: 23225, ThermoFisher). A total of 25 μg of both nuclear and cytosolic fractions of each cell line were loaded onto SDS–polyacrylamide gels. After protein separation by electrophoresis, gels were electro‐transferred onto polyvinylidene difluoride (PVDF) membranes and blocked with 5% bovine serum albumin (BSA) for 1 h at room temperature. Antibody incubations against SMAD3 (Cat. No: 9523, Cell Signaling, Danvers, MA, USA, 1 : 1000 dilution in 5% BSA) and its phosphorylated form (S423‐S425, Cat. No: ab52903, Abcam, Cambridge, UK, 1 : 2000 dilution in 5% BSA) were performed overnight at 4 °C. In order to accurately confirm the subcellular fractions' purity, antibody incubations against nucleolin (Cat. No: ab129200, Abcam, 1 : 8000 in 5% BSA) and GAPDH (Cat. No: ab181602, Abcam, 1 : 8000 in 5% BSA) were added as loading controls for nuclear and cytosolic extracts, respectively. Subsequent incubations with the corresponding anti‐rabbit HRP conjugated secondary antibody (Cat. No: AP307P, EMD Millipore, Burlington, MA, USA, 1 : 8000 in 5% BSA) were performed. Specific antibody signal was determined with the Odyssey–FC (LI–COR) system, and subsequent image quantifications were analysed with the imagej software (v. 1.53, NIH, Bethesda, MD, USA).

### 2.11 Single‐cell analyses

Validation of candidate genes was performed using recent data from a glioblastoma stem cell single‐cell analysis generated by Richards et al. [44]. Processed scRNA‐seq count data and metadata with GBM subtype annotations were used for the recreation of the scRNA‐seq objects using seurat (v.4.1.1) [45]. We discarded low‐quality cells with fewer than 200 genes detected, cells with a mitochondrial content above 5%, cells with extremely high coverage of RNA counts (> 10 000) and lowly expressed genes that were detected in less than three samples. We selected *IDH* ^WT^ patient‐derived GBM stem cell samples cultured using adherent conditions (*n* = 13). Gene expression normalization was performed with the LogNormalize function in seurat using a scaling factor of 10 000. Dimensionality reduction was conducted using a PCA on all expressed genes from the Injury Response or the Developmental GBM stem cell states, as defined by Richards et al. [44]. To reduce the contribution of the donor in subsequent analyses, sample‐to‐sample variability was corrected using harmony (v.0.1.0) [46]. Significant components were used as inputs for nonlinear dimensionality reduction techniques (UMAP) and a total of 12 significant principal components, determined by the inflection point using the scree plot method, were used for dimensional reduction purposes.

### 2.12 Pharmacological interventions and cell viability assays

Pharmacological experiments on the pd‐GBSC lines U3117‐PN, U3073‐MS and U3056‐CL, were performed using the following small molecules: T‐5224 (Cat. No: HY‐12270, MedChem, Monmouth Junction, NJ, USA), SIS3 (Cat. No: 566405, Calbiochem, Darmstadt, Germany), temozolomide (Cat. No: T2577, Merck, Darmstadt, Germany), Ro5‐3335 (Cat. No: HY‐108470, MedChem) and CADD522 (Cat. No: HY‐107999, Medchem). T‐5224 specifically inhibits the DNA‐binding activity of c‐Fos/c‐Jun (AP‐1 complex) without affecting other transcription factors [47]. SIS3 attenuates the transforming growth factor (TGF)‐β1‐induced phosphorylation of Smad3 [48]. Temozolomide is an alkylating/methylating agent that damages the DNA and triggers the death of tumour cells [49]. The benzodiazepine Ro5‐3335 interacts with RUNX1 and CBFβ and represses RUNX1/CBFB‐dependent transactivation [50]. CADD522 is a potent inhibitor of the DNA binding activity of RUNX proteins, but it exerts a more potent inhibition in the context of RUNX2 [51]. Cells were seeded in 96‐well plates in sextuplicate at a density of 6 × 10^3^ cells using 100 μL supplemented DMEM/F12 as described above. The IC_50_ was calculated for each cell line by adding drugs to the cells 24 h after seeding at given concentrations and 2‐fold serial dilutions. Pharmacological assays were performed individually or in combination with temozolomide on each subtype. Cell viability was measured using CellTiter‐Blue^®^ Cell Viability Assay (Cat. No: G808B, Promega, Madison, WI, USA) according to the manufacturer's instructions. Fluorescence at 530(25) Ex/590(35) Em was measured with the automated microtiter plate reader Synergy HT (BioTek, Winooski, VT, USA), and in order to reduce well‐to‐well variability, a row‐wise normalization approach was performed prior to statistical calculations using the ComBat function from the r/bioconductor package sva (v.3.38.0).

### 2.13 Statistical analyses

Statistical analyses were performed using r programming language and publicly available software from the r/bioconductor and cran repositories. Details on the statistical methods used in this work (MOFA framework, RNA‐seq, DNA methylation, region set enrichment analyses, elmer and Pharmacological interventions) are provided in the corresponding Methods section and in the figure legends of the manuscript.

## 3 Results

### 3.1 Multi‐omics factor analysis unveils potential sources of variability in patients with GBM

**Fig. 1:**
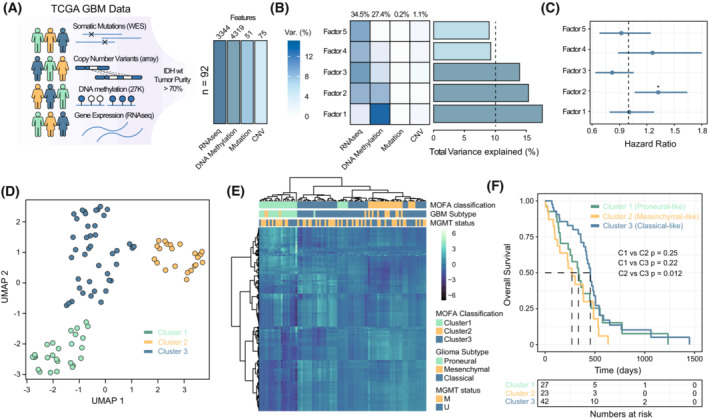
Multi‐omics factor analysis identifies distinct clusters associated with genetic, epigenetic and transcriptional alterations in GBM. (A) Schema depicting the selection criteria for the inclusion of TCGA–GBM samples. On the right, barplots indicating the total number of informative features selected from the various ‐omic layers: transcriptomic, epigenomic, genomic (whole exome sequencing, WES) and copy number variation (CNV), which were included in the multi‐omics factor analysis (MOFA) of 92 TGCA GBM patients (*IDH* ^WT^ – Wild type for isocitrate dehydrogenase mutations). (B) Heatmap illustrating the percentage of total variance explained by each of the omic layers in each of the top 5 factors identified by MOFA. On the right, the barplot depicts the cumulative variance contribution of each factor regardless of the ‐omic type analysed. The percentage of total variance explained by each independent ‐omic type is indicated at the top. (C) Line plot indicating the Cox‐proportional hazard results between the survival time of the patients and the different factors identified by MOFA. Asterisk denotes statistical significance of the Cox proportional‐hazards model (*: *P*‐value < 0.05) and lines indicate the confidence interval (CI) of each comparison. (D) Uniform Manifold Approximation and Projection (UMAP) representation of patients clustered according to the information contained in the top 3 factors identified by MOFA. Cluster imputations of the different patients were performed using a spatial clustering approach (see Section 2). (E) Heatmap illustrating the content of the 372 most informative gene expression‐DNA methylation features obtained from the MOFA analysis. The data shows the imputation of TCGA–GBM samples in different clusters using either the MOFA‐based or the GlioVis classification approach. M and U indicate the hyper‐ or hypomethylation status of the methylguanine methyltransferase (*MGMT*) gene, respectively. (F) The Kaplan–Meier plots represent the survival estimates of the different clusters identified by MOFA. *P*‐value refers to differences in event rates between the Kaplan–Meier curves and was calculated with the Log‐rank test function.

GBM comprises a highly heterogeneous set of tumours whose prognostic status significantly differs across GBM molecular subtypes. However, these classifications have been mostly derived from single‐omics data, such as whole‐genome sequencing, exome‐seq or RNA‐seq approaches [8, 10]. Considering the potential implications of the genomic, epigenomic and transcriptional layers in the final molecular outcome, we performed a multi‐omics factor analysis to integrate this information from a multi‐modal perspective and identify latent factors that might explain the major sources of variability in the patient group with GBM. We applied MOFA, a recent multi‐omic analysis framework [21], in order to combine information relating to the most variable features identified in the context of somatic mutations, CNVs, gene expression and DNA methylation status across 95 patients with GBM from the TCGA–GBM dataset (Fig. 1A). After model optimization, MOFA identified five latent orthogonal factors with a minimum explained variance of 5% in at least one or more data types (Fig. 1B, Figs S1 and S2A). Factors 1, 3 and 4 were associated with the segregation of the different glioma subtypes described by Wang et al. [10], while factors 2 and 5 were related to tumour purity (Fig. S2B) and the methylation status of the *MGMT* promoter displayed a significant association with factor 1. Interestingly, factor 2 was significantly associated with a worse survival outcome of patients with GBM (Fig. 1C) and factors 1, 2 and 3, which each independently explained more than 10% of the variance of the dataset (Fig. 1B), were more strongly implicated in the establishment of the different GBM subtypes (Fig. S2C).

With respect to the genomic contribution involved in the observed variability in these three highly informative factors, we observed that factor 1 was aligned with the somatic mutation status of the *PDFGRA*, *TP53* and *PIKCA* genes, while factor 2 was dominated by *NF1*, *PTEN* and *RB1* mutations and factor 3 was enriched in *TP53* alterations and associated with an underrepresentation of *EGFR* mutations (Fig. S2D). In relation to the alterations in the copy number, we observed a variable gain or loss of weight depending on the factors studied, being of greater relevance the relationships with alterations in chromosome 4 (*CASP3*, *FBXW7*) in the context of factor 1, alterations in chromosomes 5 and 10 (*SVIL*, *FGFR4*) in the context of factor 2 and the negative relationships found in the context of factor 3 and chromosome 6 (*QKI*) and chromosome 9 (*CDKN2A*; Fig. S2E). Using a spatial clustering approach based on the values of these three factors, we identified three patient subsets on the basis of their combined molecular profiles (Fig. 1D), which have a high degree of similarity with previous GBM classification systems (Fig. 1E, cluster1 ~ PN, cluster 2 ~ MS and cluster 3 ~ CL), each subset exhibiting different survival outcomes (Fig. 1F), with the MS‐like subtype (cluster 2) being the most aggressive in terms of prognosis.

### 3.2 Identification of transcriptional alterations in GBM reveals major molecular pathways involved in the phenotypic differentiation of GBM subtypes

**Fig. 2:**
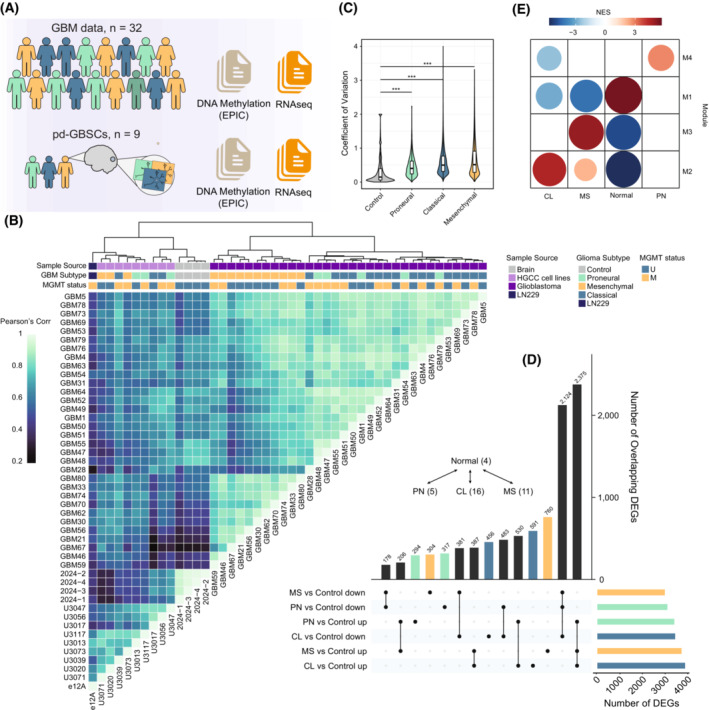
Identification of Differentially Expressed Genes (DEGs) and molecular pathways between GBM subtypes. (A) Schema showing the number of samples obtained from publicly available databases (GSE119774 and GSE119834) or pd‐GBSC lines from the Human Glioblastoma Cell Culture (HGCC) resource. (B) Heatmap indicating the pairwise Pearson's correlations at the level of gene expression (RNA‐seq) between non‐tumoral cells, GBM cells or patient‐derived GBM stem cells (pd‐GBSCs) analysed in this work. Clustering of the samples was performed using the Ward.D method. Imputation of glioblastoma subtypes was obtained using the resulting RNA‐seq data in the GlioVis portal and the human GBM cell line LN229 was included for control purposes. Imputation of the *MGMT* promoter methylation status (U: unmethylated; M: methylated) was obtained using the R package mgmtstp27 (C) Violin plots indicating the coefficient of variation at the gene expression level calculated for the different primary bulk GBM subtypes. Asterisks denote statistical significance between cancer and control groups by means of a Wilcoxon Rank‐sum test (***: *P*‐value <0.001). (D) UpSetR plot representing the total number of differentially expressed genes (horizontal bars) and the potential overlaps (vertical bars) between non‐tumoral brain and the different GBM subtypes (adj. *P*‐value < 10^−6^). The number of samples and the number of differentially expressed genes (DEGs) identified in each of the comparisons are indicated. (E) Bubble plot illustrating the normalized enrichment scores of the different modules identified in the modular co‐expression analyses and the non‐tumoral tissue or Proneural (PN), Classical (CL) or Mesenchymal (MS) subtypes used in this work. Red and blue indicate, respectively, enrichment or underrepresentation of each of the modules in a given GBM subtype.

Considering the advantages of new DNA methylation microarray technologies (EPIC vs. 27 K/450 K) and the next‐generation sequencing platforms that have emerged in recent years, we were encouraged to perform an in‐depth exploration of the different ‐omic layers in a bid to identify relevant molecular pathways involved in GBM stratification in additional cohorts. We generated gene expression and DNA methylation data from a set of pd‐GBSCs obtained from the Human Glioblastoma Cell Culture resource (HGCC) and complemented with additional bulk GBM patient data obtained from a recent methylome‐transcriptome analysis performed by Mack et al. [17] (Fig. 2A). Hierarchical clustering of patients with GBM and pd‐GBSC samples, based on pairwise gene expression correlations using the GBM gene signature derived by Wang et al. [10], revealed the presence of two clearly defined clusters, one dominated by a MS‐like subtype and the other by CL‐PN‐like subtypes (Fig. 2B). The different GBM subtypes displayed larger expression variability as compared with the control brains, both in the context of primary GBMs (Fig. 2C) and pd‐GBSCs (Fig. S3A), which is consistent with the gene expression heterogeneity arising from the cancer context. Of note, this variability between subtypes was less pronounced in the context of pd‐GBSCs compared to primary GBMs, suggesting a possible influence of cell culture conditions on the maintenance of intrinsic gene expression patterns corresponding to the different tumour subtypes. We next performed a differential expression analysis of PN, CL and MS subtypes compared with non‐tumoural brain tissue. Our pipeline identified 6507, 7327 and 6715 DEGs in the context of PN, CL and MS subtypes, respectively (Fig. 2D). An analogous analysis in the context of non‐matched pd‐GBSCs revealed, respectively, 4377, 3905 and 4706 DEGs for the same subtypes (Fig. S3B), with the overlap between primary GBM samples and their corresponding GBM stem cell lines being greater than 50% considering the DEGs identified when both sample groups are compared (Fig. S3C, PN OR = 1.9, *P*‐value < 0.001; CL OR = 1.9, *P*‐value < 0.001; MS OR = 1.9, *P*‐value < 0.001).

In order to explore the subtype‐specific transcriptional rewiring of GBM samples, we performed a gene co‐expression network analysis using the 1000 most variable genes from the RNA‐seq data, and found four different gene expression modules (Fig. 2E). Module 1 was enriched in non‐tumoural brain tissue, while module 2, module 3 and module 4 were mainly dominated by the CL, MS and PN subtypes, respectively (Fig. 2E). Interestingly, we observed a differential gene set enrichment signature for each of these modules that was consistent with their known biological features. Module 1 was enriched in gene ontologies related to neural system development and synaptic regulation, in accordance with the functions exerted by the brain cortex (Fig. S4). Module 2 enrichments were mainly related to epigenetic regulation and G2M checkpoint, while module 3 was enriched in extracellular matrix components, immune responses and the epithelial to MS transition, the latter being consistent with the phenotype observed in the GBM MS subtype. Enrichments in module 4 were associated with axon development and the differentiation of oligodendrocytes, suggesting a closer relationship with the PN subtype (Fig. S4). These results confirm that the subtype‐specific transcriptional rewiring observed in the context of cancer governs the phenotypic diversity observed in patients with GBM.

### 3.3 Differential DNA methylation patterns disclose molecular footprints associated with the different GBM subtypes

**Fig. 3:**
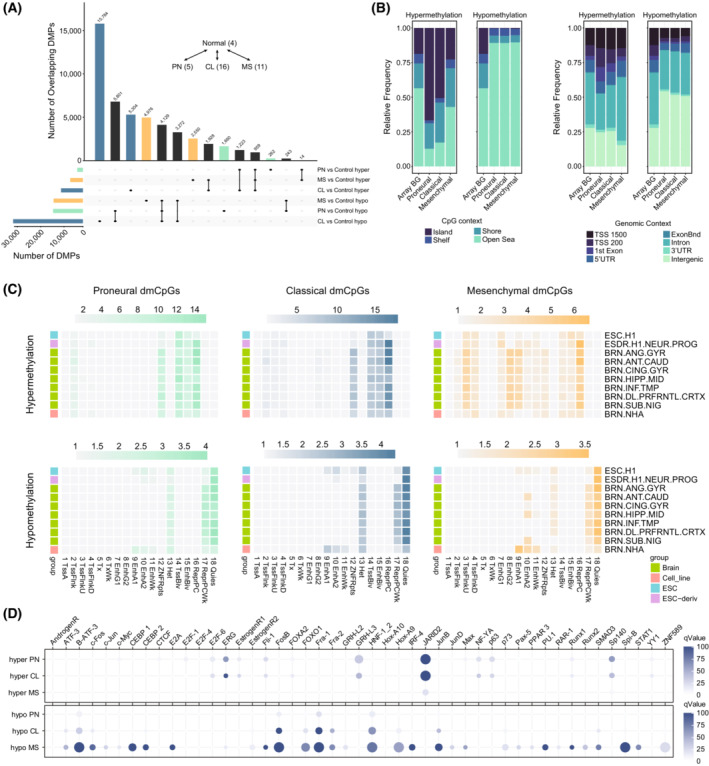
DNA methylation footprint of GBM subtypes discloses specific TFBS signatures during tumour transformation. (A) UpSetR plot representing the total number of differentially methylated probes (DMPs, horizontal bars) and their potential overlaps (vertical bars) between non‐tumoural brain and the different GBM subtypes (adj. *P*‐value < 0.05, *B*‐Value > 0.3). The number of samples and the number of DMPs identified in each of the comparisons are indicated. (B) Stacked barplots indicating the relative frequency of significant hyper‐ or hypomethylated CpGs in relation to their CpG context (left) or CpG location (right). (C) Heatmaps depicting chromatin state enrichment analyses of hyper‐ and hypomethylated CpGs which are common or specific to the different GBM subtypes as compared to non‐tumoural brain tissue. Colour scales indicate the odds ratio (OR) of significant DMPs from previous analyses across 18 chromatin states obtained from the NIH Roadmap Epigenome consortium as compared with the background distribution of the Human Methylation EPIC platform. (D) Bubble plots showing enrichment of transcription factor binding sites (TFBSs) in the indicated conditions as determined by the information obtained from the gene transcription regulation database (GTRD). Bubble colour denotes statistical significance (−Log_10_ adj. *P*‐value) and dot size indicates the Log_2_ OR enrichment of a particular TFBS dataset as compared with the background distribution of the EPIC platform.

The epigenomic landscape is closely related to alterations in the transcriptional layer and has revealed important clues regarding the molecular heterogeneity of GBM from a different perspective [17, 52]. Interestingly, the DNA methylation layer contributed significantly to explaining the heterogeneity of GBM samples as determined by our MOFA analysis. Thus, to identify additional molecular aspects involved in the phenotypic diversity of GBM, we performed a differential DNA methylation analysis comparing non‐tumoural brain tissue with PN, MS and CL GBM subtypes. We found a total of 15 291, 39 400 and 18 071 DMPs, respectively, in the context of the PN, CL and MS subtypes in primary GBMs and, again respectively, 30 413, 28 623 and 37 515 DMPs in pd‐GBSCs (Fig. 3A, Fig. S5A). The number of hypomethylated DMPs outperformed the number of hypermethylated DMPs in all the comparisons, suggesting a global loss of DNA methylation during the tumourigenic process. It is worth noting that, in general, pd‐GBSCs showed a greater number of alterations than their GBM patient counterparts, and their subsequent overlaps ranged from 13% to 55% of the total number of DMPs identified in the pd‐GBSC set (Fig. S5B, PN OR = 12.3, *P*‐value < 0.001; CL OR = 11.1, *P*‐value < 0.001; MS OR = 5.6, *P*‐value < 0.001), indicating a potential effect of cell culture conditions on the epigenome in the context of these pd‐GBSCs. A detailed inspection of the genomic context of these DMPs indicated an enrichment of hypermethylated CpG sites at CpG islands and regions proximal to promoter locations, irrespective of GBM subtype, with the enrichments being of greater magnitude in the context of the DMPs specific to the PN and CL subtypes (Fig. 3B, Fig. S5C). In contrast, loss of DNA methylation was observed at open sea locations and intergenic regions and showed subtle differences between the PN and CL GBM subtypes.

To explore the potential implications of these epigenomic alterations in GBM, we performed a comprehensive region set enrichment analysis based on chromatin segmentation data from 10 tissue types including healthy brain tissue and embryonic stem cells obtained from the Roadmap Epigenomics Consortium [40]. These analyses revealed substantial differences in the chromatin context of the PN, CL and MS GBM subtypes (Fig. 3C). DNA hypomethylation was enriched in heterochromatin and quiescent states in a subtype‐independent manner (Fig. 3C, Fig. S6A), as would be expected from the observed genomic context of these CpG sites. In contrast, hypermethylated DMPs were generally associated with bivalent enhancers and transcription start sites, zinc fingers and a repressive Polycomb state in normal tissues, independent of GBM subtype (Fig. 3C, Fig. S6A). Intriguingly, DNA hypermethylation of MS‐like samples evidenced significant enrichment at flanking transcription start sites and enhancer elements as compared to the PN and CL subtypes, and these effects were particularly noticeable in the context of primary GBM samples, though not in pd‐GBSCs (Fig. 3C).

In order to predict the involvement of potential transcription factors in the remodelled epigenomic landscape of the different GBM subtypes, we performed a TFBS enrichment analysis using information from the Gene Transcription Regulation Database (GTRD) [41]. Hypermethylated DMPs displayed significant enrichment in Polycomb‐related factors (JARID2) and the transcription factors, SP140 and GRHL3, both in the context of primary GBM samples and pd‐GBSCs (Fig. 3D, Fig. S6B). Interestingly, we identified a set of subtype‐specific enrichments in TFBS in the context of hypomethylated DMPs. These enrichments were preferentially associated with the MS‐like subtype and revealed that the transcription factors BATF3, members of the AP‐1 complex (FOS, FOSB, FRA1‐FOSL1 and FRA2‐FOSL2, JUNB), members of the CEBP family (CEBPA, CEBPB), HOXA9, SMAD3 and RUNX‐related factors (RUNX1, RUNX2) played an important role in the modulation of the epigenomic landscape of the most aggressive GBM subtype (Fig. 3D). Most of these observations were also observed in pd‐GBSCs (Fig. S6B), indicating that the expression or the activity of these transcription factors exert a subtype‐specific footprint in the epigenome, and suggests that they could probably be exploited as potential molecular vulnerabilities of GBM in the context of personalized medicine.

### 3.4 Integrative DNA methylation – gene expression approach reveals the presence of subtype‐specific molecular vulnerabilities

**Fig. 4:**
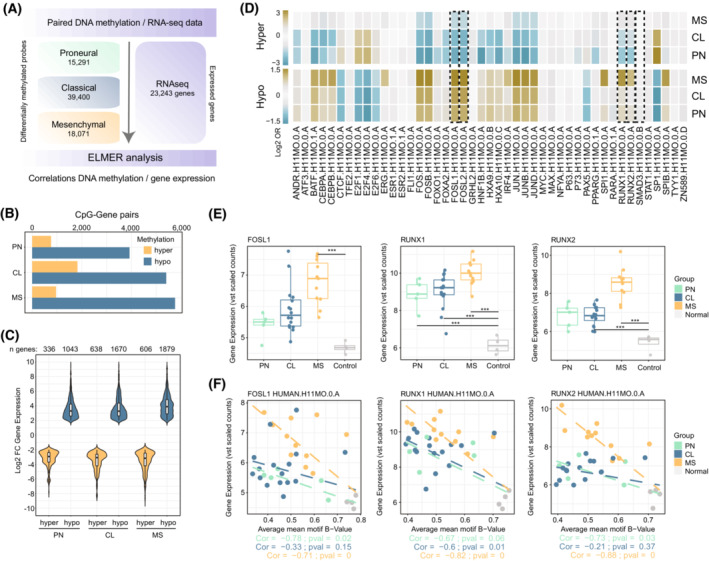
Integrative DNA methylation – Gene expression analysis reveals subtype‐specific vulnerabilities in GBM. (A) Schema depicting the number of subtype‐specific differentially methylated probes (DMPs) and the number of genes used in the DNA methylation‐gene expression approach. (B) Barplot illustrating the total number of significant DMP‐gene pair correlations identified using the Enhancer Linking by Methylation/Expression Relationships (elmer) algorithm in each of the GBM subtypes. For ease of interpretation, data is segregated according to the methylation status of the DMP (hyper‐ or hypomethylated). (C) Violin plot reflecting the differential expression status (tumour vs. non‐tumour) of the genes displaying strong DNA methylation‐gene expression correlations in the context of the subtype‐specific DMP‐gene pairs. The number of genes included in each of the comparisons is noted at the top of the column. (D) Heatmap indicating the enrichment of transcription factor binding sites (TFBS; Log_2_ OR) using the significant hyper‐ or hypomethylated probes from the elmer approach. Dashed lines are positioned over transcription factors which displayed substantial differences between GBM subtypes and were explored in further detail. (E) Boxplots illustrating the gene expression levels of the selected transcription factors in the set of GBM samples. The upper and lower whisker lines include observations included within 1.5 times the interquartile range, while points outside this range represent outliers. For interpretation purposes, samples were divided according to their initial imputed GBM subtype. Asterisks denote statistical significance between the different categories obtained from the DESeq2 analysis (***: adj. *P*‐value < 10^−6^). (F) Scatter plot showing the Spearman correlation between average DNA methylation of the indicated transcription factor (TF) motif targets including the expression levels of the regulatory TF. Patients are represented by dots and are coloured according to the GBM subtype.

In light of the aforementioned results, and in order to gain further insights into the relationships between the transcriptional and the epigenetic layers, we used elmer [42] to integrate paired DNA methylation and RNA‐seq data from these patients with GBM. elmer correlations were calculated using DMPs identified in the different primary GBM samples and all the genes that had been expressed in at least one condition from the RNA‐seq data (Fig. 4A). For biological interpretation purposes, we focused on those correlations supported by the classical model of epigenetic regulation, and we observed a total of 4685, 7232 and 6722 significant CpG‐gene pair correlations in the context, respectively, of the PN, CL and MS GBM subtypes (Fig. 4B). In a similar vein, DNA hypermethylation was mainly associated with reduced gene expression levels, while DNA hypomethylation was correlated with gene activation (Fig. 4C). We next performed a detailed TFBS analysis considering those gene expression‐correlating DMPs using publicly available human TF binding models from the HOCOMOCO database [53]. These analyses validated our previous epigenomic observations and revealed the significant enrichment of AP‐1 members (FOSL1–FOSL2) and RUNX factors (RUNX1–RUNX2), both of which were more evident in the context of the MS phenotype (Fig. 4D). We observed a significant variation in gene expression levels of these factors depending on the GBM subtype involved (Fig. 4E), and the average DNA methylation status of their DNA‐binding motifs substantially decreased as a consequence of the increased gene expression level of these transcription factors in the context of the MS, but not the PN or CL GBM subtypes (Fig. 4F). It is worth noting that we did not observe a significant anti‐correlation between the gene expression levels of *SMAD3* or *CEBPs* and their potential effect on the modulation of correlated DMP‐gene pairs (Fig. 4D, Fig. S7A,B) despite the significant enrichment displayed in the previous epigenomic analysis. This suggests that additional layers of gene regulation, such as post‐translational modifications, may be involved in the establishment of the observed epigenomic footprints mentioned above. Indeed, in the case of SMAD3, we observed that these effects may be mediated by increased protein stability and nuclear localisation mediated by the phosphorylated form of the protein (Fig. S7C).

To discard the potential contribution of cell type heterogeneity in the identification of the molecular vulnerabilities from bulk RNA‐seq data and to corroborate whether those alterations were intrinsic to GBM stem cells, we validated our observations with data from a recent single‐cell RNA‐seq study in the context of patient‐derived GBM stem cells [44]. The present work identified two clusters, one related to immunomesenchymal properties and the other to developmental/PN properties, based on the intrinsic expression profiles of these GBM stem cells, similar to features observed in the MS and PN subtypes, respectively (Fig. S8). We validated that both GBM stem cell subtypes expressed the stem cell marker *SOX2* as well as subtype‐specific markers, such as *OLIG2* and *BAALC* (PN) or *CD44* (MS‐like subtype) (Fig. S8C,D). In addition, we observed the significant differential expression of *AP‐1* and *RUNX*‐related members, with their gene expression levels being more pronounced in the context of the MS‐like phenotype (Fig. S8D,E). These observations were also confirmed in the context of our pd‐GBSC data (Fig. S8F), indicating that most of the observed molecular vulnerabilities are intrinsic to GBM stem cells.

### 3.5 Pharmacological inhibition of AP‐1, SMAD3 or RUNX1/RUNX2 restrains the tumourigenic potential of GBM stem cell lines

**Fig. 5:**
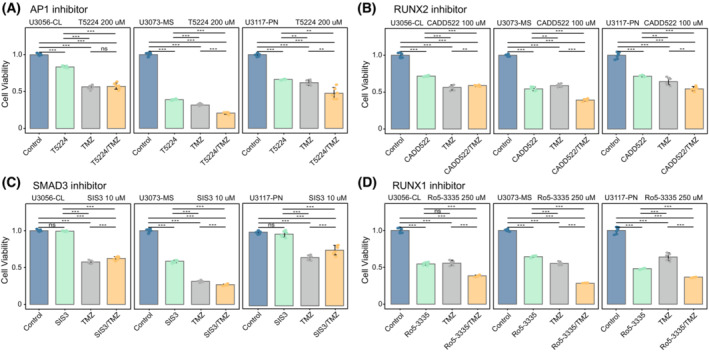
Pharmacological modulation of pd‐GBSCs confirms the subtype‐specific effects observed in previous integration approaches. (A–D) Barplots representing the cell viability status of a Proneural (PN), a Classical (CL) and a Mesenchymal (MS) patient‐derived GBM stem cell (pd‐GBSCs) subjected to pharmacological inhibition of AP‐1 with the chemical compound T‐5224 (A), RUNX2 inhibition with the small drug CADD522 (B), SMAD3 inhibition with the SIS3 reagent (C) and RUNX1 inhibition using the compound Ro5‐3335 (D). The concentration of the different pharmacological interventions is indicated at the top of each graph, and the concentration of the combined treatment with temozolomide or temozolomide alone was kept at a constant concentration of 800 μm. All the conditions were assayed at least in quintuplicate, error bars represent the standard deviation (SD) and asterisks denote statistical significance obtained from a Welch's *t*‐test between the indicated comparisons (ns, not‐significant, **: *P*‐value < 0.01, ***: *P*‐value < 0.001).

To explore whether targeting the TFs mentioned above represents a therapeutic opportunity in GBM, we performed the pharmacological inhibition of AP‐1, RUNX1 and RUNX2 with the respective available chemical inhibitors T‐5224, Ro5‐3335 and CADD522. In addition, considering the overall impact of SMAD3 on the methylome of the MS subtype despite the lack of significant changes at the level of gene expression, we also performed the pharmacological inhibition of this factor using the small molecule SIS3. Optimal drug concentrations were determined using a dose‐dependent assay and IC_50_ approach. As expected, inhibition of AP‐1 at a fixed dose was more evident in the context of the MS pd‐GBSCs (Fig. 5A), although pharmacological inhibition of RUNX2 and SMAD3 also impaired cell viability in the MS subtype to a greater extent than in the PN and CL subtypes (Fig. 5B,C). In contrast, no significant differences were observed between GBM subtypes in the context of RUNX1 inhibition (Fig. 5D). To validate the efficacy of these pharmacological approaches, the inhibitors involved were combined with the GBM standard‐of‐care treatment based on temozolomide at a fixed dose. Combinations of small drug inhibitors with temozolomide impaired cell viability in all the pd‐GBSCs, but this impairment was more evident in the context of the MS subtype compared to their PN and CL counterparts (Fig. 5A–D), thus confirming the role of these molecular vulnerabilities in sustaining the tumourigenic potential of MS‐like GBM stem cells.

## 4 Discussion

The incorporation of molecular approaches to cancer treatment has led to an improvement in personalized medicine, enabling the identification of specific molecular alterations that result in better segregation and classification of patients which results in improved disease management. In this study, we applied different multi‐omic approaches, such as methylome and transcriptome profiling, to deal with the inherent GBM heterogeneity, which has allowed us to distinguish various GBM subtypes with particular molecular characteristics that closely resemble previous classifications [10, 11, 12]. These approaches proved useful in determining the contribution of particular signalling pathways that may sustain the tumourigenic capacities of the different GBM subtypes, with substantial differences reflected between MS compared to the PN and CL subtypes, in line with previous reports that linked the MS subtype with immune system infiltration and overexpression of microglia‐associated genes [10, 54].

In addition, we found that the epigenomic layer captured different sources of heterogeneity, both in the context of the different glioblastoma subtypes and also between glioblastoma stem cells and their tumour tissue source, in a similar fashion to that described by Mack et al. [17]. Despite the global loss of DNA methylation in GBM described here and in other studies [14], this heterogeneity was mostly explained in the context of DNA hypermethylation, with well‐defined differences among subtypes in the context of genomic enrichments and subrogated chromatin states. A gain in hypermethylation in the MS subtype predominantly occurred outside the CpG island context and was associated with flanking transcription start sites and enhancer elements in non‐tumoural tissues as compared to their PN and CL counterparts. These discrepancies may arise from the greater number of cell types and histological heterogeneity observed in the MS subtype, but may also reflect a potential subrogated effect in the tumour microenvironment as regards the establishment of an epigenetic immunoediting programme to elicit immune evasion, as has recently been described by Gangoso et al. [55]. In relation to the observations obtained in the context of pd‐GBSCs, there are also intrinsic experimental limitations that must be taken into account. On the one hand, the results obtained in the comparison with non‐tumoural brains may contain alterations related to the effect of cell culture conditions, leading to a potential normalization of subtype‐specific alterations and masking potential enrichments in relevant factors or molecular pathways involved in GBM. On the other hand, the establishment of cell culture conditions in stem cell medium may influence the cellular metabolism of the different subtypes of pd‐GBSCs, leading to potential cellular tolerance or addiction and the consequent normalization of the pathways associated with each subtype. Therefore, our strategy focused on exploring those common alterations observed in both GBM and pd‐GBSCs, in order to identify those alterations consistent in GBM samples.

Despite these limitations, the DNA methylation layer proved to be a valuable tool for the identification of molecular footprints associated with the activation of particular TF programmes in GBM. Our analyses revealed the prominent effect of several TFs such as B‐ATF‐3, members of the AP‐1 complex (FOSB, FRA‐1‐FOSL1, FRA‐2‐FOSL2, JUNB), RUNX factors (RUNX1/RUNX2) and SMAD3, particularly in the context of the MS subtype. These transcription factors orchestrate a transcriptional rewiring that favours the development of different GBM programmes, for example, FOSL1 has recently been described as a key regulator of the MS subtype and governs an intrinsic MS gene signature in GBM stem cells that promotes an immune MS phenotype [44, 56, 57]. The transcription factor FOSL2 is associated with the hypoxic status of the tumour [58] and has been related to the extent of necrosis in GBM samples and as governing a MS transcriptional signature in GBM [59]. With regards to RUNX‐related factors, RUNX1 has recently been implicated in the regulation of an anti‐apoptotic programme in GBM [60] and contributes to the MS subtype in a TGF‐B‐dependent manner [61]. In addition, RUNX2 promotes malignant progression in glioma [62] and maintains MS tissue homeostasis through IGF signalling [63]. Surprisingly, despite the lack of correlation between SMAD3 gene expression levels and the epigenetic modulation of its cognate putative binding sites, we observed the pronounced effect of this factor in the methylome of the MS subtype, suggesting that either the expression or the activation of TFs by additional regulatory mechanisms, such as post‐translational modifications, may be responsible for the observed outcome. Interestingly, SMAD proteins are activated in a TGF‐B‐dependent manner [64], and some of the heterogeneity of GBM subtypes may be explained by particular post‐translational modifications, such as phosphorylation and palmytoilation [65, 66], which modify SMAD3 activity and may promote the transcriptional rewiring that leads to a MS‐like phenotype.

Consistent with the prominent role of these TFs in GBM identified in our analyses, therapeutical targeting of the AP‐1 complex, RUNX1, RUNX2 or SMAD3 factors alone or in combination with the canonical GBM chemotherapeutic agent temozolomide displayed a subtype‐specific impairment on cell proliferation with greater effects in the context of the MS subtype. Altogether, the combination of multi‐omics data may provide a better classification of GBM patients into clinically relevant subgroups and facilitates the identification of molecular vulnerabilities that may be amenable to the rational design of novel therapeutic strategies in GBM.

## 5 Conclusions

In this study, we have identified subtype‐specific molecular vulnerabilities of GBM by integrating transcriptomic and epigenomic data from bulk GBM and pd‐GBSCs. We demonstrate that therapeutical intervention on the transcriptional pathways mediated by AP‐1, SMAD3 and RUNX1/RUNX2, combined with the GBM standard‐of‐care treatment based on temozolomide, led to the pronounced impairment of tumour growth, which was more evident in the context of the MS‐like subtype. These results emphasize the use of multi‐omic approaches for the discovery of cancer cell vulnerabilities and point towards the crucial role of these factors in the development of GBM.

## Author contributions

PS‐O, JRT, AFF and MFF conceived the study and designed the experiments. PS‐O, VL, AR and CM performed the experiments. PS‐O, JRT and RFP performed the computational analyses and analysed the data. PS‐O, JRT, AFF and MFF wrote the manuscript. All the authors approved the manuscript.

## Conflict of interest

The authors declare no conflict of interest.

### Peer review

The peer review history for this article is available at https://www.webofscience.com/api/gateway/wos/peer‐review/10.1002/1878‐0261.13479.

## Supporting information


**Fig. S1.** Optimization of multi‐omics factor analysis models.

**Fig. S2.** Clinical correlations observed in the factors identified by the multi‐omics factor analysis model.

**Fig. S3.** Differential Gene Expression analyses of Glioblastoma Stem Cells.

**Fig. S4.** Gene sets and molecular pathways enriched in the different Glioblastoma clusters identified in the co‐expression analysis approach.

**Fig. S5.** Differential Methylation changes observed in Glioblastoma Stem Cells.

**Fig. S6.** Chromatin state and TFBS enrichments on pd‐GBSC data.

**Fig. S7.** Correlation between SMAD3 and CEBPs expression levels and the epigenetic status of their cognate putative binding sites.

**Fig. S8.** Validation of candidate TF using single‐cell Glioblastoma Stem Cell data.

**Data S1.** Legends.

## Data Availability

Raw HumanMethylationEPIC array data including IDAT files of pd‐GBSCs lines and non‐tumoural brains have been deposited in Array Express under the accession number (E‐MTAB‐12473). Raw RNA sequencing FASTQ files of GBSCs have been deposited in the European Genome‐Phenome Archive under the accession number (EGAD00001009762). Additional DNA methylation and RNA seq datasets were obtained from Mack et al. [17] through the Gene Expression Omnibus database (GSE119774 and GSE119834). Single‐cell data was obtained from the study of Richards et al. [44] through the Broad Institute Single‐Cell Portal (SCP503). For further exploratory purposes, processed data and supplementary tables have been deposited in the Zenodo repository under the accession number 10.5281/zenodo.7380252.
