# Supplementary figures and images for: Multi‐omic integration of DNA methylation and gene expression data reveals molecular vulnerabilities in glioblastoma
Source: Mol Oncol. 2023 Jul 20;17(9):1726–43. doi: 10.1002/1878-0261.13479 (PMC10483606; doi:10.1002/1878-0261.13479)

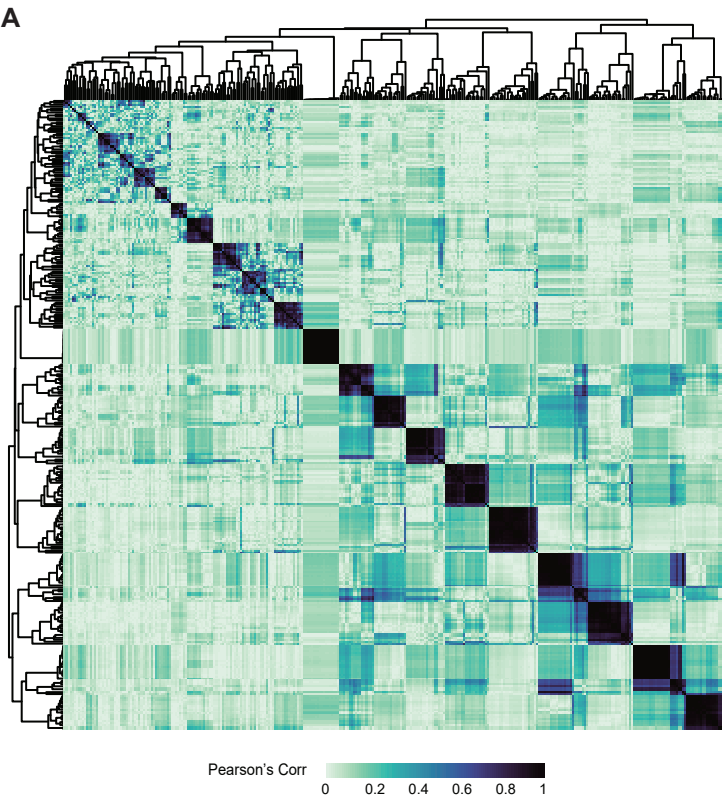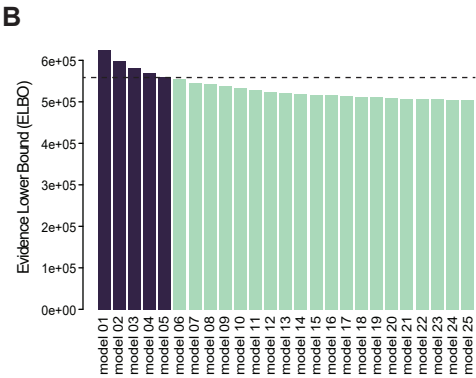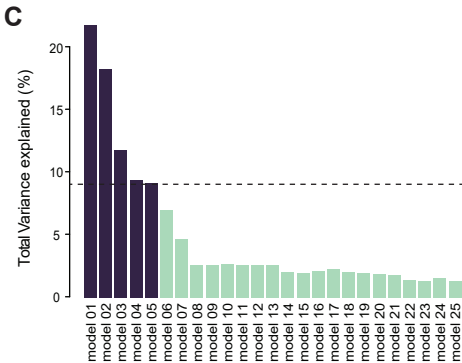

Supplement: Supplementary file 1 — Fig. S1. Optimization of multi‐omics factor analysis models. [file MOL2-17-1726-s005.pdf]

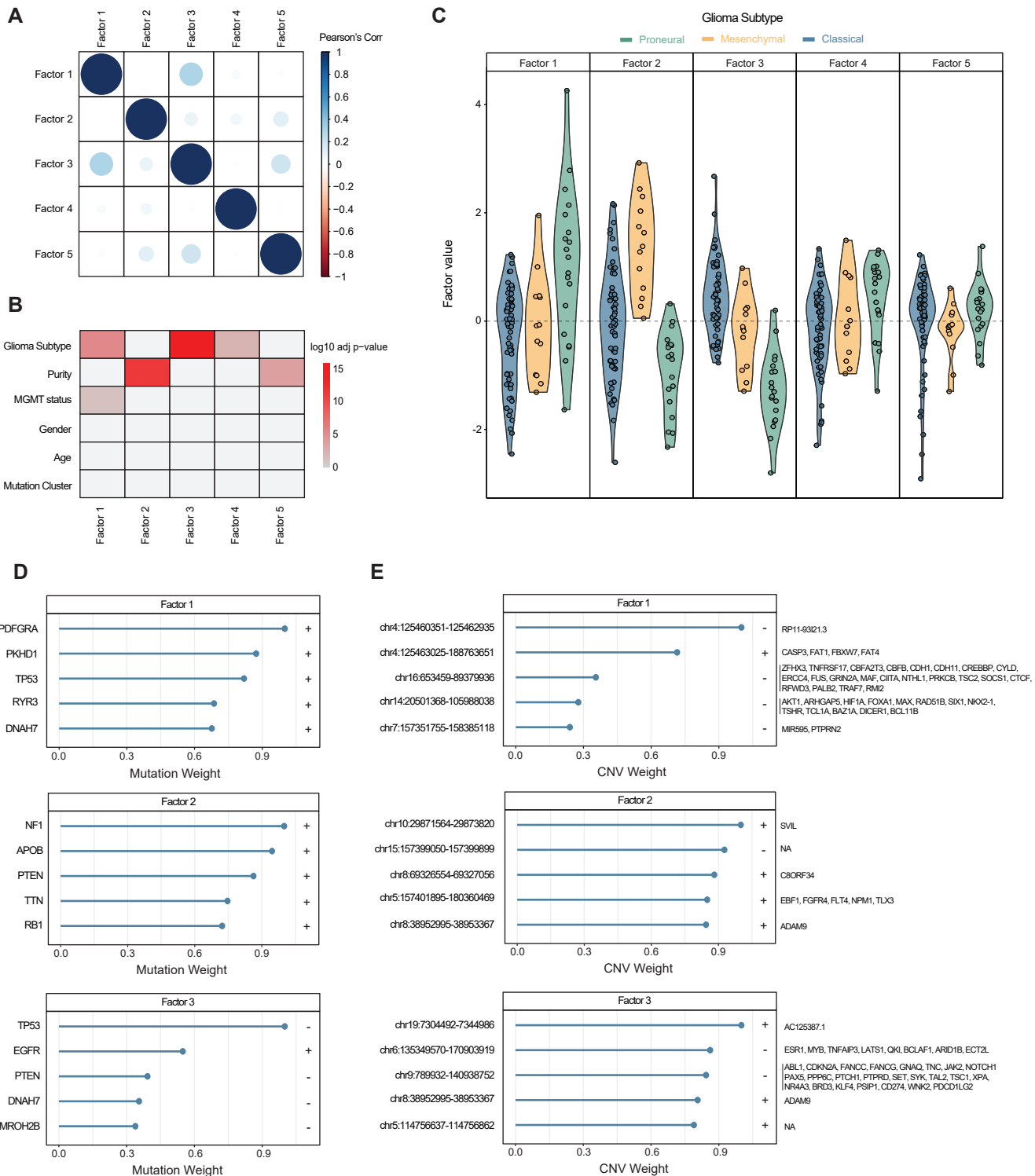

Supplement: Supplementary file 2 — Fig. S2. Clinical correlations observed in the factors identified by the multi‐omics factor analysis model. [file MOL2-17-1726-s008.pdf]

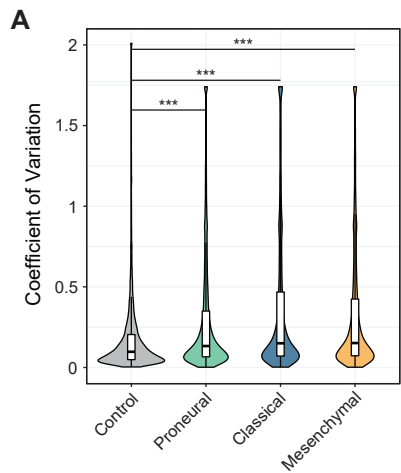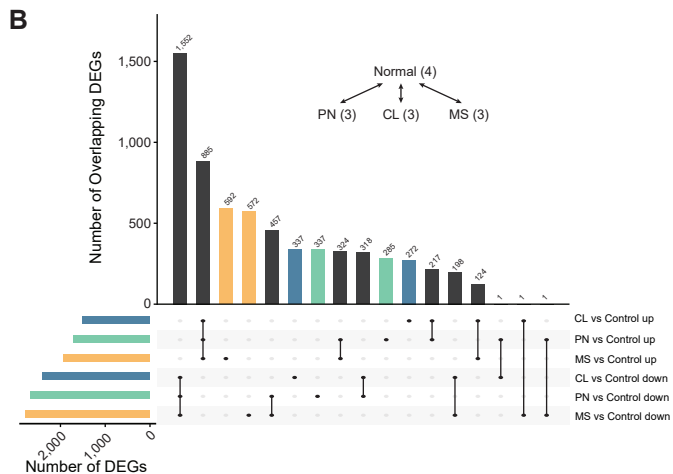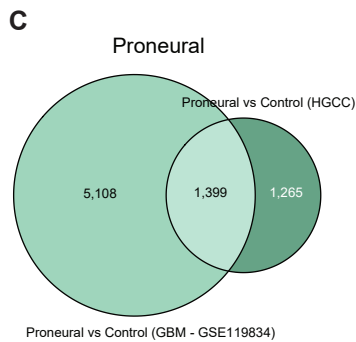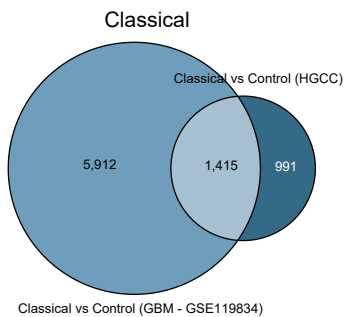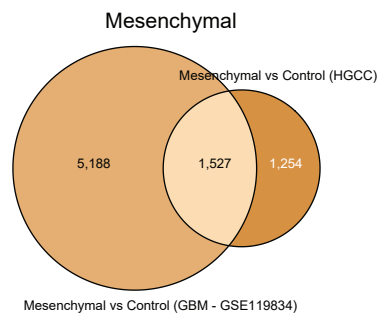

Supplement: Supplementary file 3 — Fig. S3. Differential Gene Expression analyses of Glioblastoma Stem Cells. [file MOL2-17-1726-s002.pdf]

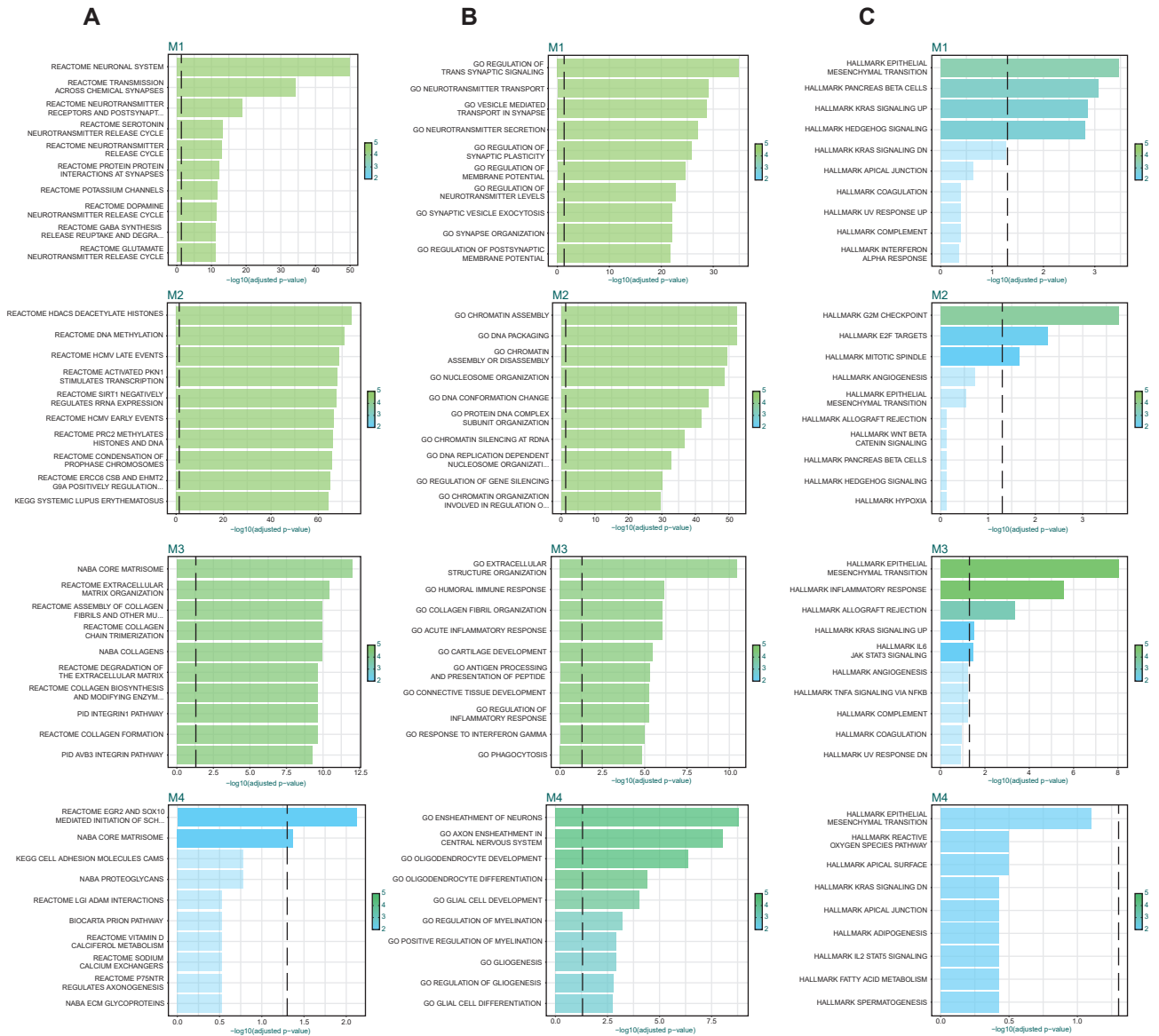

Supplement: Supplementary file 4 — Fig. S4. Gene sets and molecular pathways enriched in the different Glioblastoma clusters identified in the co‐expression analysis approach. [file MOL2-17-1726-s009.pdf]

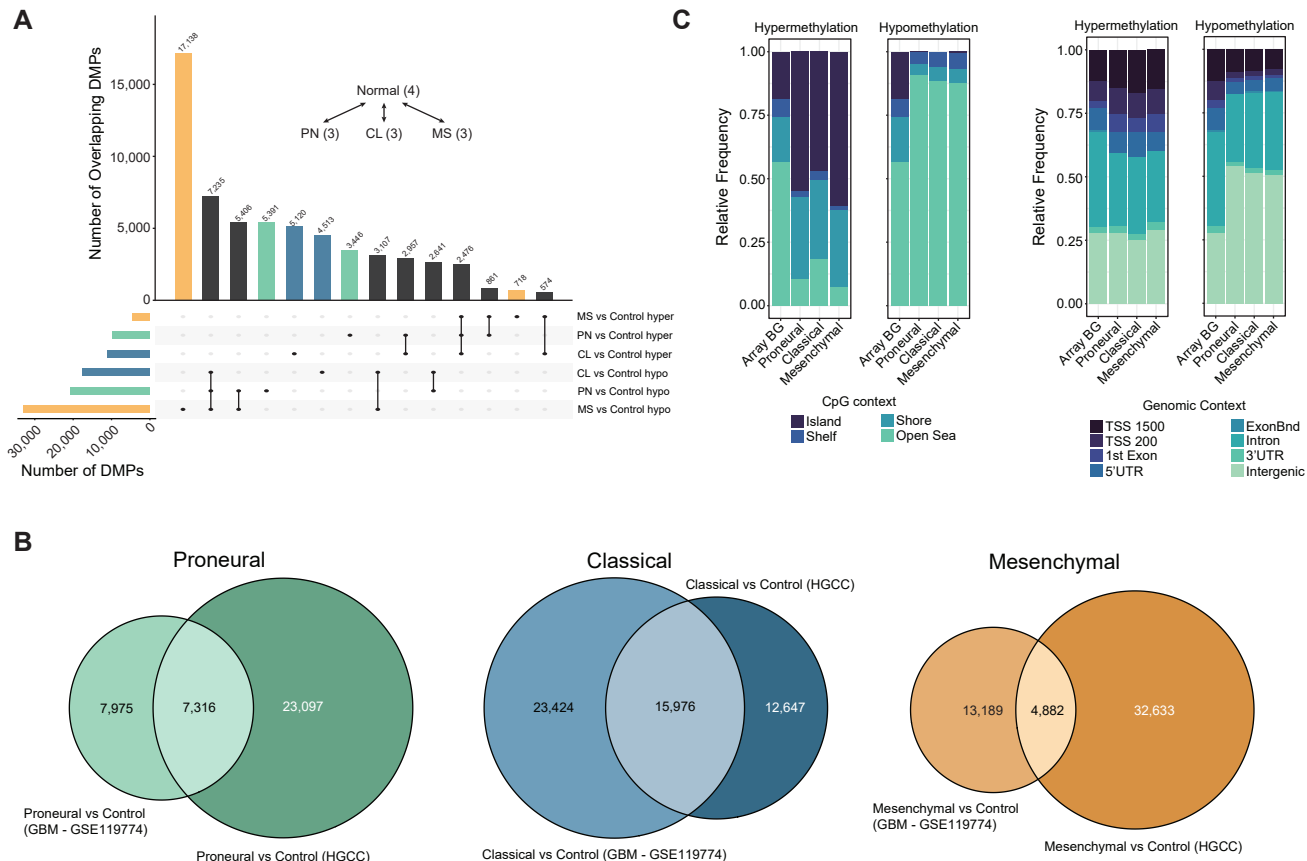

Supplement: Supplementary file 5 — Fig. S5. Differential Methylation changes observed in Glioblastoma Stem Cells. [file MOL2-17-1726-s007.pdf]

**A**

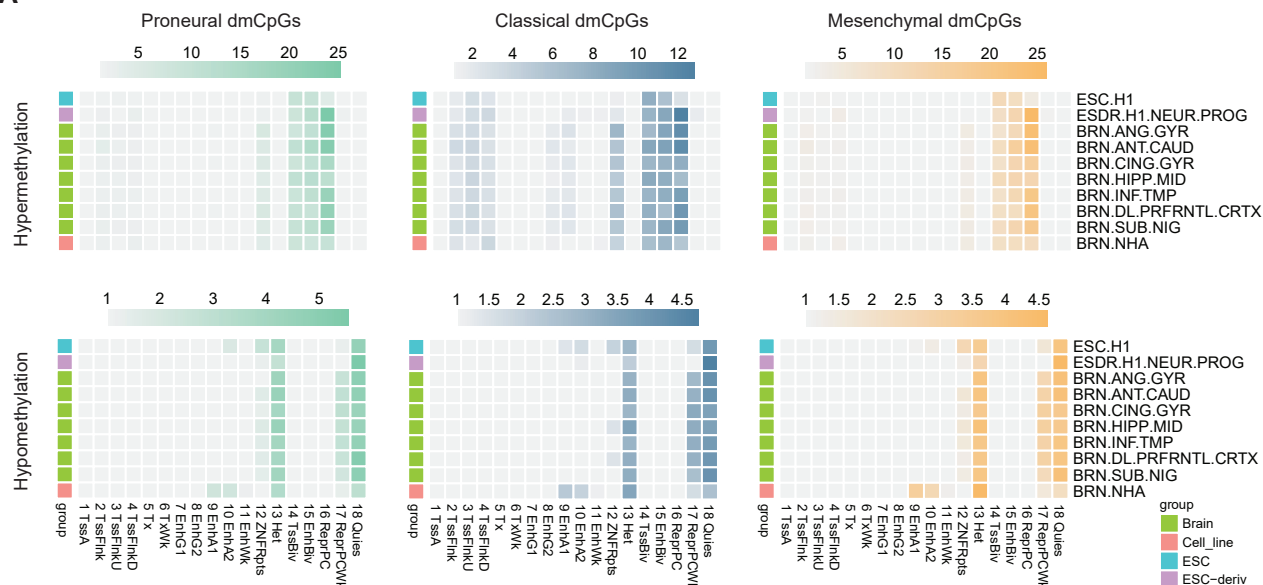

**B**

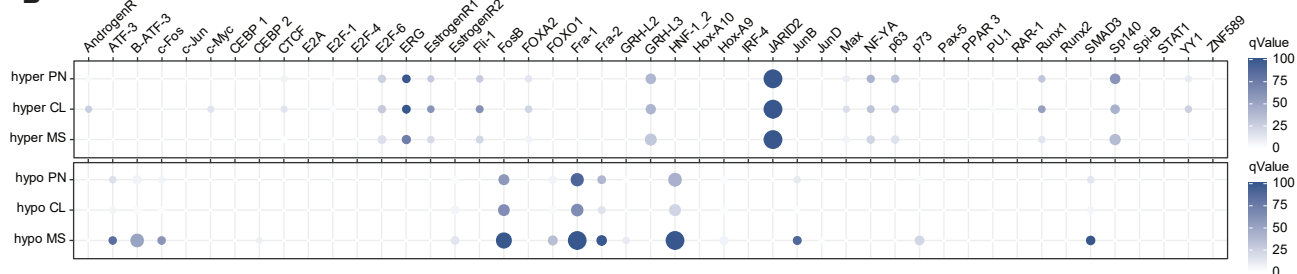

Supplement: Supplementary file 6 — Fig. S6. Chromatin state and TFBS enrichments on pd‐GBSC data. [file MOL2-17-1726-s004.pdf]

**A**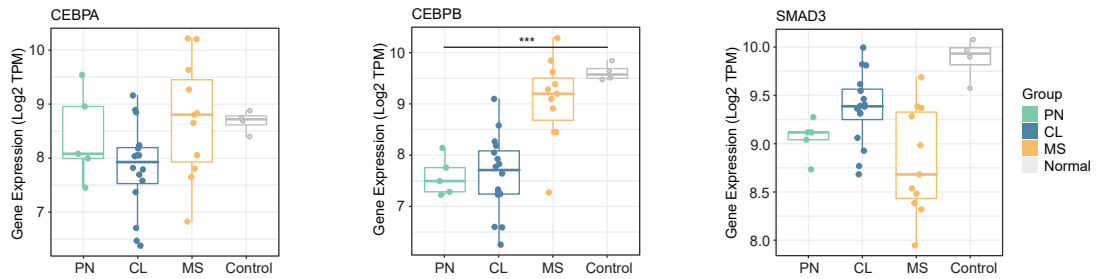**B**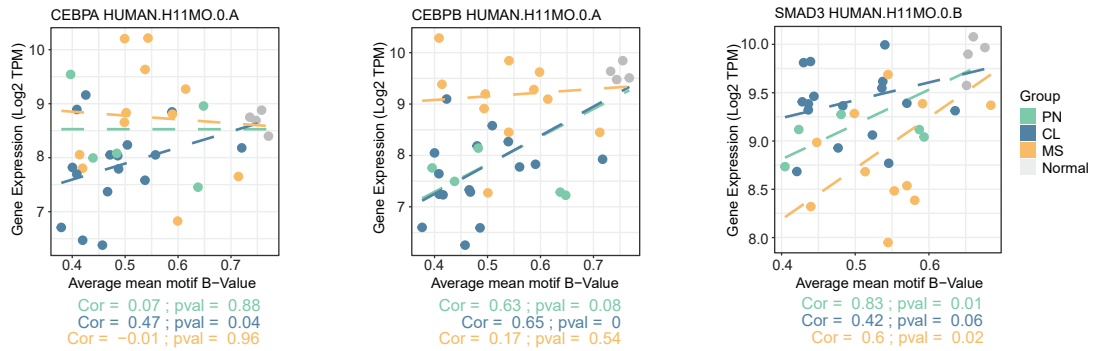**C**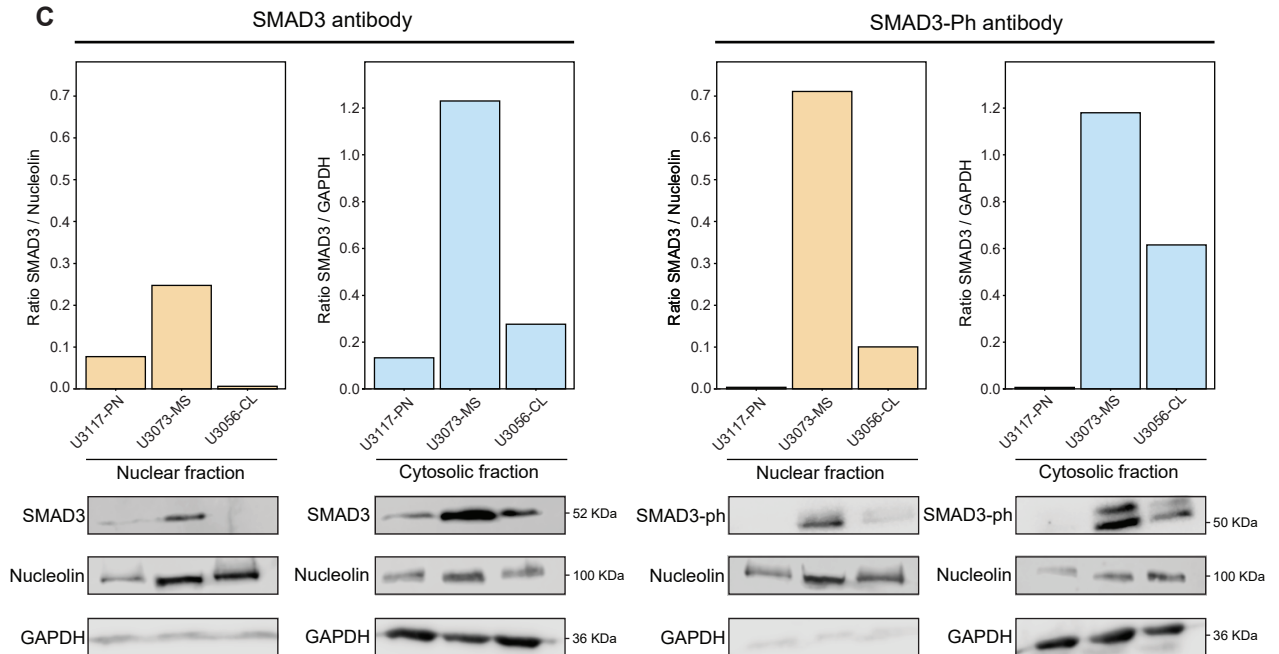

Supplement: Supplementary file 7 — Fig. S7. Correlation between SMAD3 and CEBPs expression levels and the epigenetic status of their cognate putative binding sites. [file MOL2-17-1726-s003.pdf]

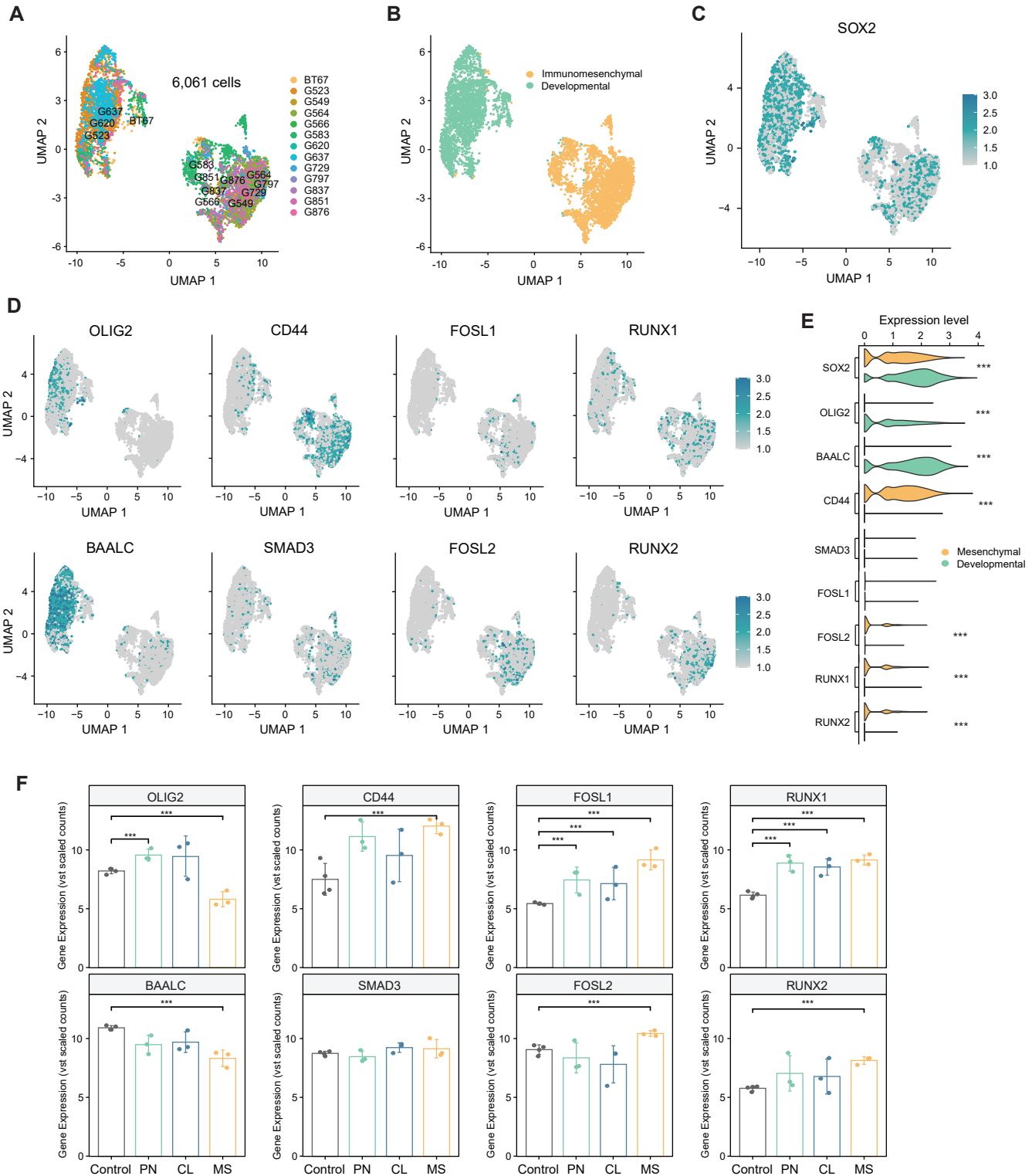

Supplement: Supplementary file 8 — Fig. S8. Validation of candidate TF using single‐cell Glioblastoma Stem Cell data. [file MOL2-17-1726-s001.pdf]
