# Supplementary material for: Multi‐omic integration of DNA methylation and gene expression data reveals molecular vulnerabilities in glioblastoma
Source: Mol Oncol. 2023 Jul 20;17(9):1726–43. doi: 10.1002/1878-0261.13479 (PMC10483606; doi:10.1002/1878-0261.13479)
Supplement: Supplementary file 9 — Data S1. Legends. [file MOL2-17-1726-s006.pdf]

SUPPORTING INFORMATION

Multi-omic integration of DNA methylation and gene expression data reveals molecular vulnerabilities in glioblastoma

Santamarina-Ojeda, Tejedor, et al., 2023

Molecular Oncology

SUPPORTING INFORMATION

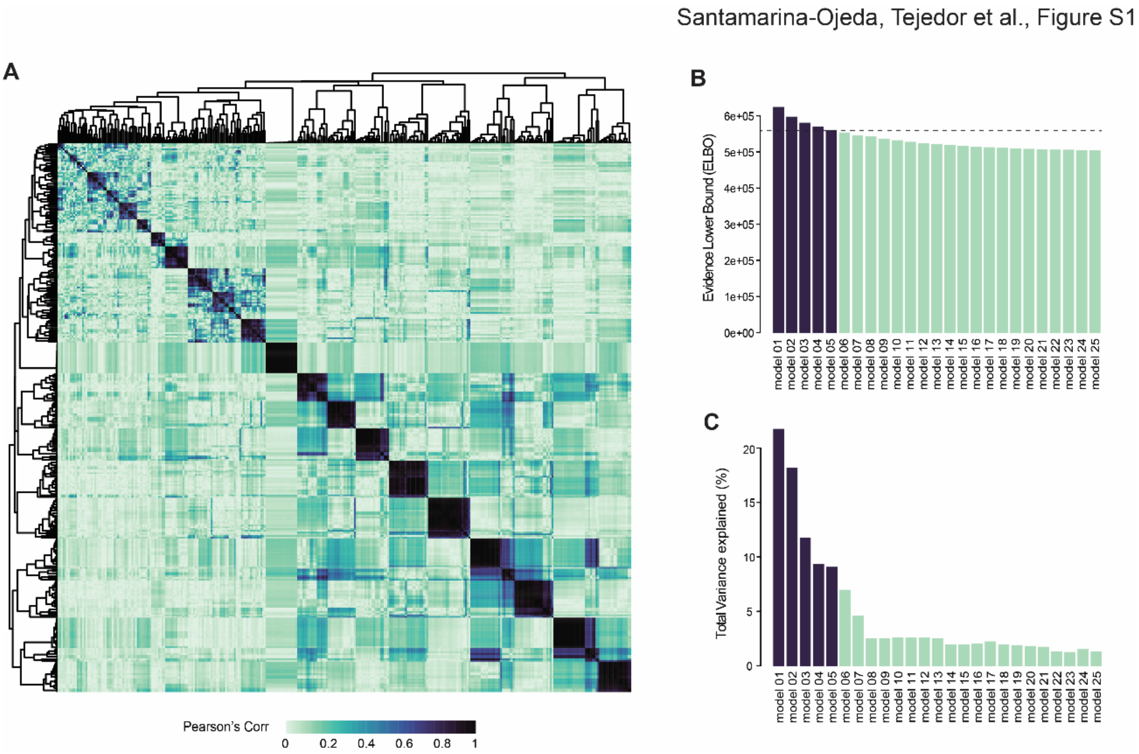

Fig. S1 – Optimization of MOFA models. (A) Heatmap representing the pairwise correlations observed between the factors studied in the different models generated, ranging from 1 to 25 factors. (B) Barplot illustrating the Evidence Lower Bound (ELBO) score estimated for each of the models generated. Each model includes an increasing number of factors (1 to 25) (C) Barplot depicting the percentage of total variance explained by each of the models generated depending on the number of factors included in each model. Dashed lines indicate the optimal cut-off as determined by the elbow approach.

Santamarina-Ojeda, Tejedor et al., Figure S2

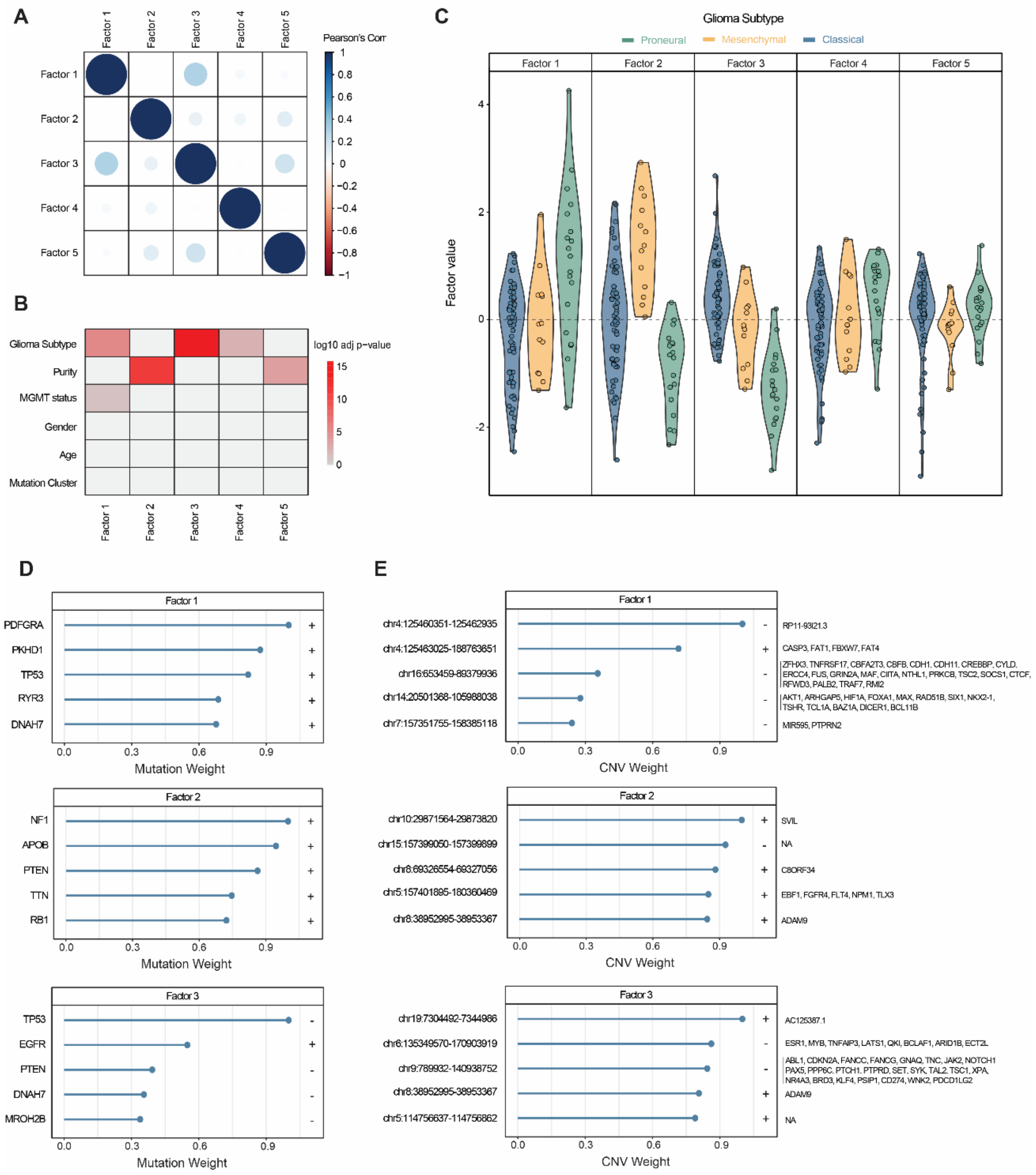

Fig. S2 – Clinical correlations observed in the factors identified by the MOFA model. (A) Scatterplot indicating the pairwise correlation observed between the different factors identified in the final MOFA model. Positive and negative correlations are denoted according to the colour scale indicated in the legend. (B) Heatmap illustrating the enrichment of the different factors in clinicopathological parameters from TCGA GBM dataset. Colour scale reflects statistical significance (-Log$_{10}$ adj. *p*-value). (C) Violin plots reflecting the factor value scores for each of the patient samples included in this analysis according to the annotated GBM subtype (proneural, classical, mesenchymal). (D) Barplots reflecting the mutation weight of the indicated GBM mutated genes in the context of each of the top 3 factors. + or – symbols indicate positive or

negative associations, respectively. (E) Same as D, but in the context of copy number variants. Only canonical driver genes included in the network of cancer genes (http://ncg.kcl.ac.uk/) have been included for visualisation purposes.

Santamarina-Ojeda, Tejedor et al., Figure S3

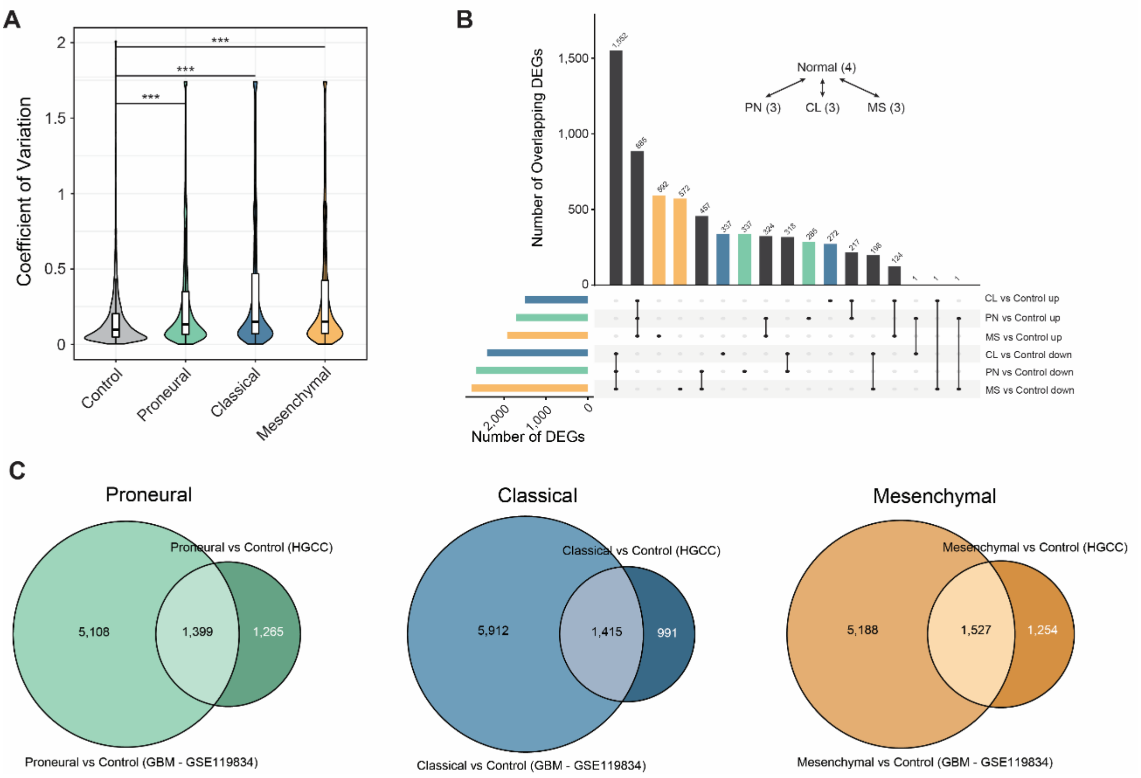

Fig. S3 – Differential Gene Expression analyses of Glioblastoma Stem Cells. (A) Violin plots reflecting the coefficient of variation at the gene expression level calculated for the different pd-GBSCs annotated according to their GBM subtypes. Asterisks denote statistical significance between cancer and control groups (***: p-value < 0.001). (B) UpSetR plot illustrating the total number of differentially expressed genes (horizontal bars) and their potential overlaps (vertical bars) between non-tumoral brain and the different pd-GBSCs studied (adj. p-value < $10e^{-6}$). The number of samples and the number of DEGs identified in each of the comparisons are indicated. (C) Venn diagrams depicting gene overlaps between DEGs identified in the GBM subtypes (Figure 2) and those identified in the pd-GBSCs analyses (HGCC).

Santamarina-Ojeda, Tejedor et al., Figure S4

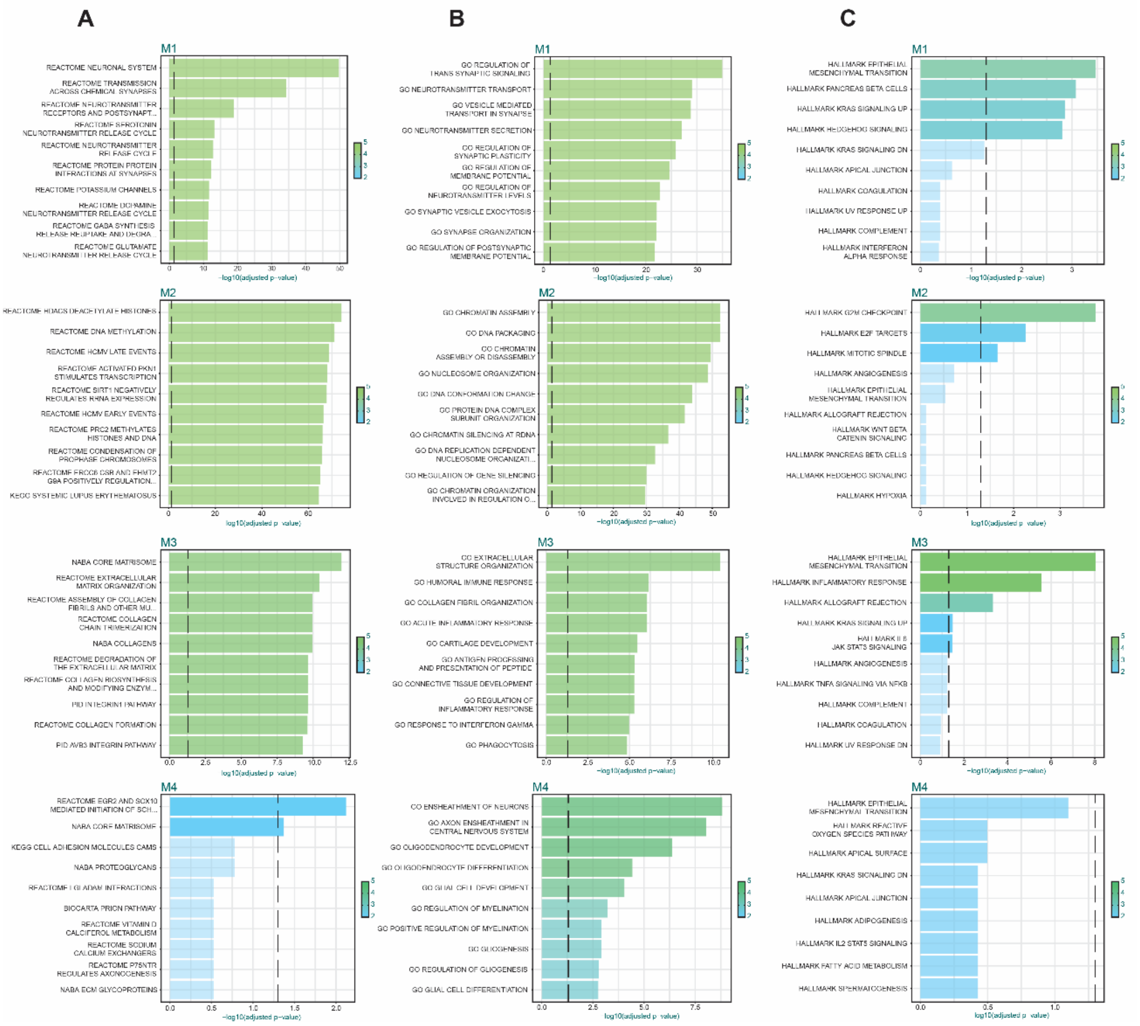

Fig. S4 – Gene sets and molecular pathways enriched in the different GBM clusters identified in the co-expression analysis approach. (A, B, C) Gene set enrichment analysis of the pathways included in the C2 (Canonical pathways, A), the C5 (Gene Ontology, B) and the Hallmark (C) MSigDB collection for genes in the different clusters (M1 to M4) identified in the coexpression analysis strategy. The length and the colour of the bar denotes the enrichment in a particular gene set category by means of the $–\mathrm{Log}_{10}$ adj. *p*-value.

Santamarina-Ojeda, Tejedor et al., Figure S5

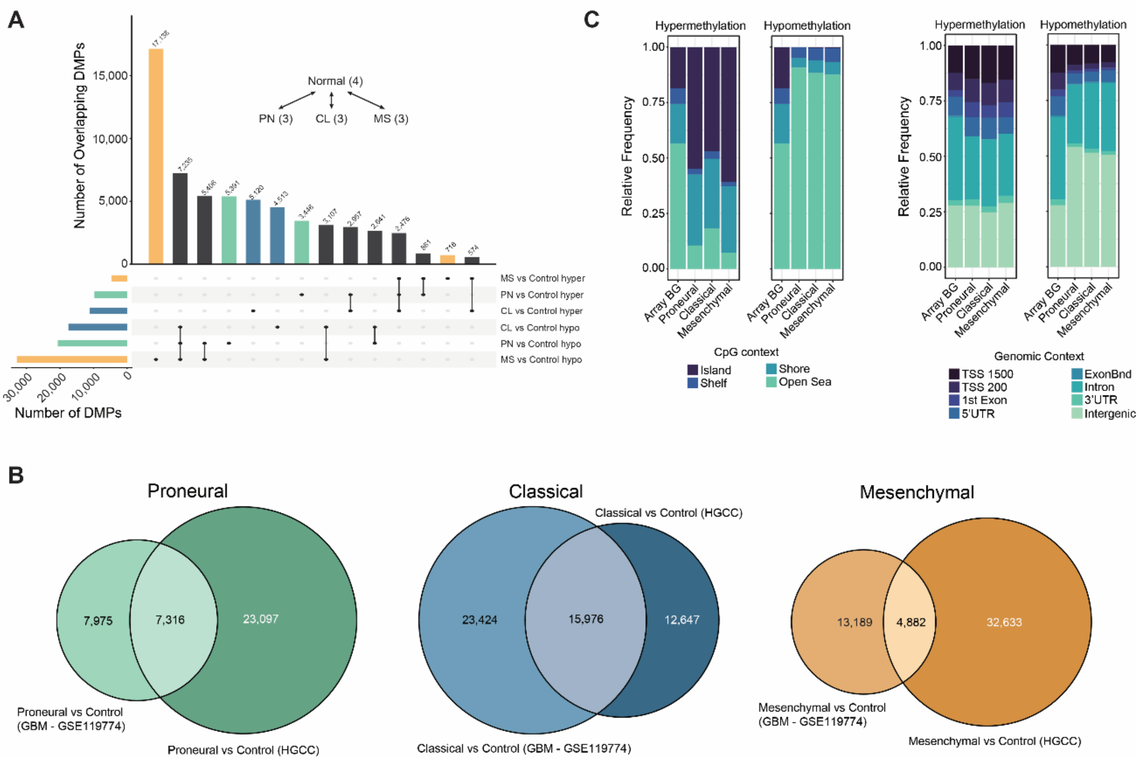

Fig. S5 – Differential Methylation changes observed in Glioblastoma Stem Cells. (A) UpSetR plot illustrating the total number of DMPs (horizontal bars) and their potential overlaps (vertical bars) between non-tumoral brain and the different pd-GBSCs studied (adj. p-value < 0.05, B-Value > 0.3). The number of samples and the number of DMPs identified in each of the comparisons are indicated. (B) Stacked barplots showing the relative frequency of significantly hyper- or hypomethylated CpGs in relation to their CpG context (left) or CpG location (right). (C) Venn diagrams depicting the DNA methylation overlaps between DMPs identified in the GBM subtypes (Figure 3) and those identified in the pd-GBSCs analyses (HGCC).

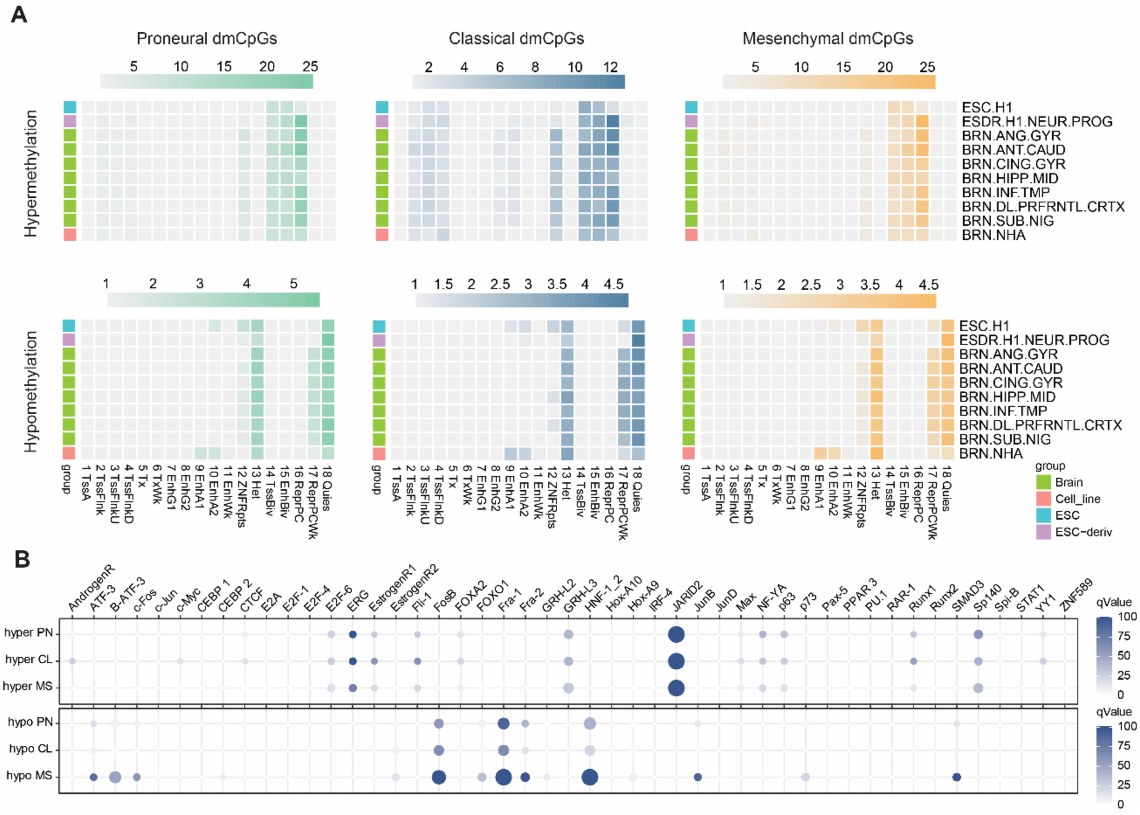

Fig. S6 – Chromatin state and TFBS enrichments on pd-GBSC data. (A) Heatmaps illustrating chromatin state enrichment analyses of hyper- and hypomethylated CpGs which are common to or specific for the different pd-GBSC lines as compared to non-tumoral brain tissue. Colour scales depict ORs of significant DMPs obtained in previous analyses across 18 chromatin states, obtained from the NIH Roadmap Epigenome consortium, as compared with the background distribution of the Human Methylation EPIC platform. (B) Bubble plots representing enrichment of TFBS in the indicated conditions as determined by the information obtained from the GTRD database. Bubble colour denotes statistical significance as compared with the background distribution of the EPIC platform.

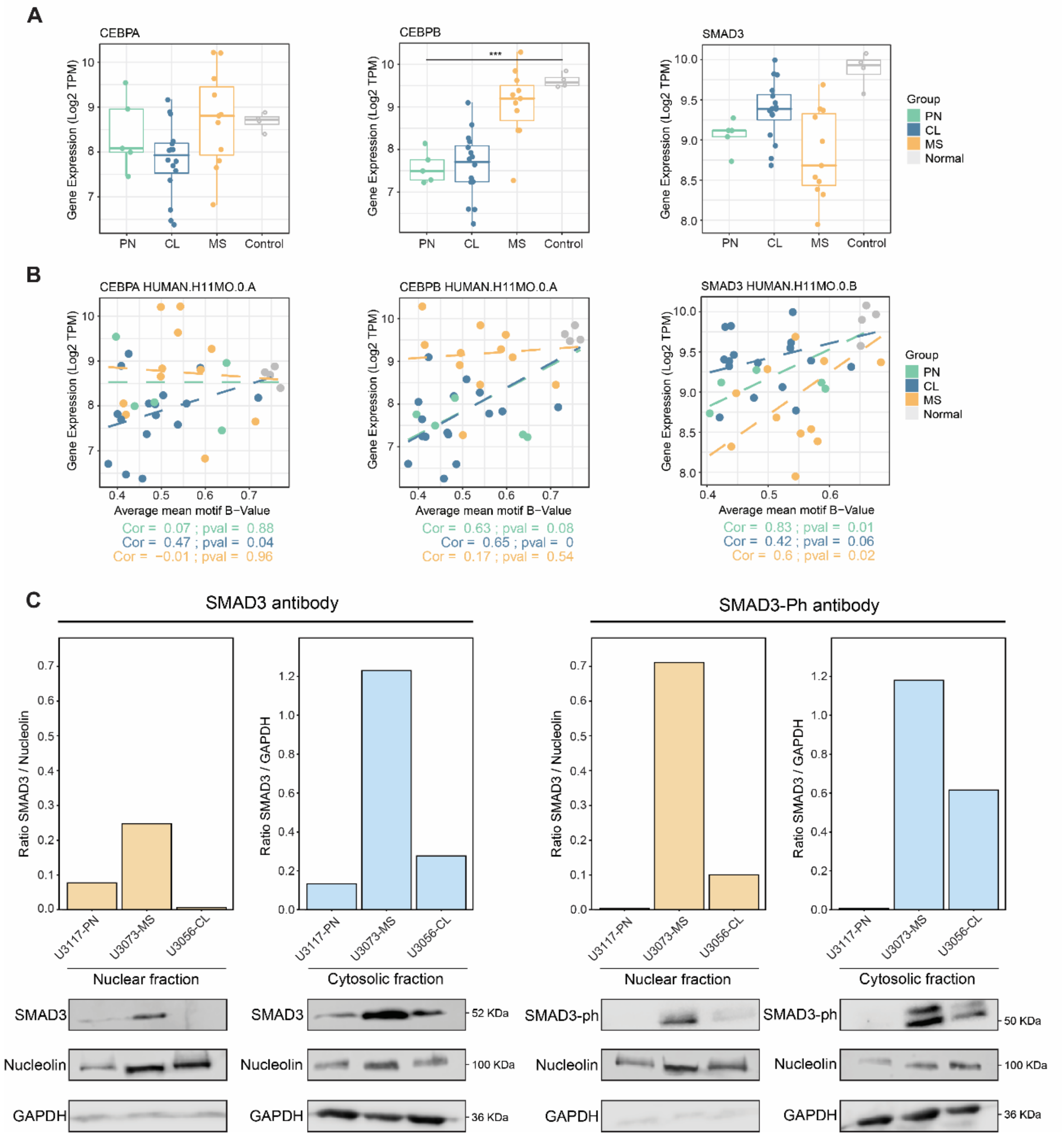

Fig. S7 – Correlation between SMAD3 and CEBPs expression levels and the epigenetic status of their cognate putative binding sites. (A) Boxplots depicting the gene expression levels of the selected transcription factors in the set of GBM samples. For interpretation purposes, samples were divided according to their initially imputed GBM subtype. Asterisks denote statistical significance between the different categories obtained from the DESeq2 analysis (***: adj. *p*-value < 10^{-6}). (B) Scatter plot showing the Spearman correlation between average DNA methylation of the indicated TF motif targets with the expression levels of the regulatory TF as in Fig 4. (C) Western blot quantification of SMAD3 or phospho-SMAD3 levels in pd-GBSCs. Blots indicate the results of the cellular fractionation protocol. Nuclear and cytoplasmic levels of SMAD3 were normalised against nucleolin (nucleus) or GAPDH (cytoplasm) levels and bar plots

indicate the relationship between SMAD3 levels and each of the abovementioned proteins in each condition.

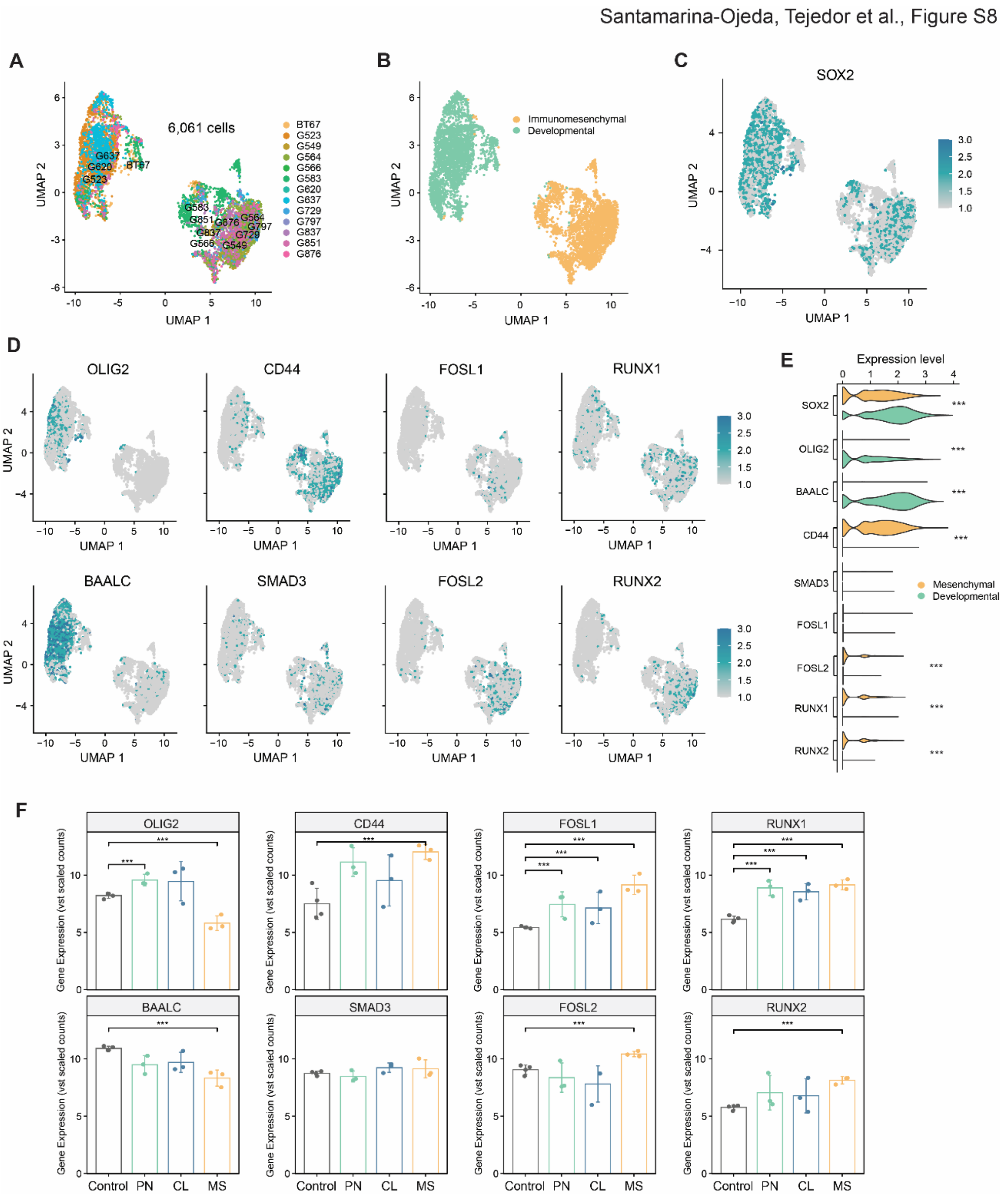

Fig. S8 – Validation of candidate TF using single cell GBM Stem Cell data. (A) UMAP representation of 6,041 GBM single stem cells derived from 13 GBM patients. Colour denotes the correspondence between the single cells and the corresponding patient. (B) Same representation as in A, but cells are coloured according to the immunomesenchymal or developmental clusters as determined by Richard and colleagues. (C) UMAP representation of

single cells. The colour indicates the expression levels of the multipotential neural stem cell marker *SOX2*. (D) Same as C, but each plot represents the expression values of the indicated TF in the single cells analysed. (E) Violin plot representing the average expression values of the indicated transcription factors on the basis of immunomesenchymal or developmental clusters. (A, B, C, D) Barplots representing the gene expression status of the indicated TFs in our pd-GBSC cohort. Asterisks denote statistical significance between the different categories obtained from the DESeq2 analysis (***: adj. p-value $< 10^{-6}$).
